# Synthesis and biological activity evaluation of 3-(hetero) arylideneindolin-2-ones as potential c-Src inhibitors

**DOI:** 10.1080/14756366.2022.2117317

**Published:** 2022-09-01

**Authors:** Salvatore Princiotto, Loana Musso, Fabrizio Manetti, Valentina Marcellini, Giovanni Maga, Emmanuele Crespan, Cecilia Perini, Nadia Zaffaroni, Giovanni Luca Beretta, Sabrina Dallavalle

**Affiliations:** a Department of Food, Environmental and Nutritional Sciences (DeFENS), University of Milan, Milan, Italy; b Dipartimento di Biotecnologie, Chimica e Farmacia, Università di Siena, Siena, Italy; c Institute of Molecular Genetics IGM, CNR “Luigi Luca Cavalli-Sforza”, Pavia, Italy; d Molecular Pharmacology Unit, Department of Applied Research and Technological Development, Fondazione IRCCS Istituto Nazionale Tumori, Milan, Italy

**Keywords:** c-Src, indolinone, Knoevenagel reaction, molecular docking

## Abstract

Inhibition of c-Src is considered one of the most studied approaches to cancer treatment, with several heterocyclic compounds approved during the last 15 years as chemotherapeutic agents. Starting from the biological evaluation of an *in-house* collection of small molecules, indolinone was selected as the most promising scaffold. In this work, several functionalised indolinones were synthesised and their inhibitory potency and cytotoxic activity were assayed. The pharmacological profile of the most active compounds, supported by molecular modelling studies, revealed that the presence of an amino group increased the affinity towards the ATP-binding site of c-Src. At the same time, bulkier derivatizations seemed to improve the interactions within the enzymatic pocket. Overall, these data represent an early stage towards the optimisation of new, easy-to-be functionalised indolinones as potential c-Src inhibitors.

## 1. Introduction

**Figure 1.**
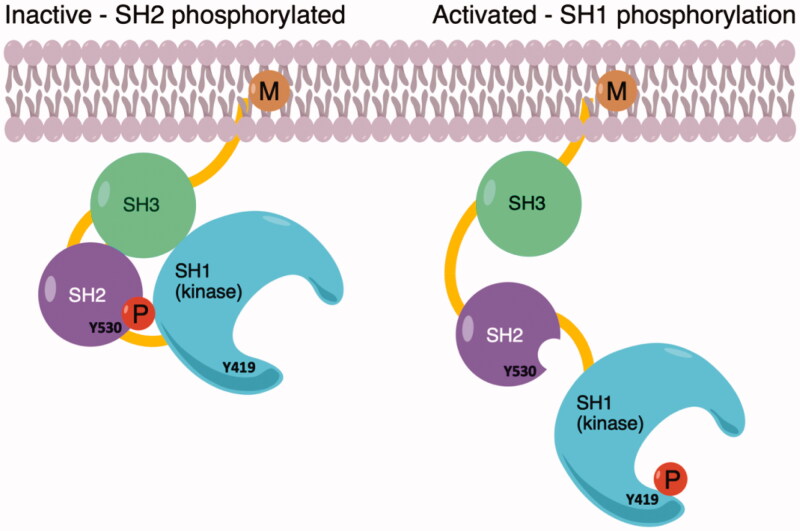
(a) Inactive conformation of c-Src shows phosphorylated Tyr530 on the SH2 domain; (b) Phosphorylation of Tyr419 on the kinase domain SH1 allows to activate of the enzyme.

c-Src is the first discovered and most studied member of the Src kinase family^1^. It is a cytoplasmic non-receptor protein kinase^2^, highly involved in intracellular signal transmission for the regulation of proliferation, migration, adhesion, invasion, and drug resistance of cancer cells^3^^,^^4^. c-Src is a 60-kDa protein anchored to the plasmatic and endosomal membranes, as well as in the nuclear compartment, through its N-terminal tail, in which basic residues are particularly abundant^5^. The protein structure consists of four domains: SH1, the kinase region in which the autophosphorylation site is present; SH2, which hosts the phosphorylated Tyr530 (in the C-terminal tail) and binds to PDGFR; SH3, responsible for the closed and inactive conformation of the protein; SH4, which presents the myristoylation site, essential for the membrane localisation^6^. c-Src is constitutively inactive, with a close conformation in which SH2 and SH3 protect a phosphorylated Tyr530, preventing interactions within the catalytic site. Dephosphorylation of Tyr530 triggers the activation of c-Src, whose open conformation allows Tyr419 phosphorylation and full availability to ligand-protein interactions^7^^,^^8^ (Figure 1).

Overexpression and/or high kinase activity of c-Src is associated with cancer progression and metastasis processes, mainly because of the reduced cell-cell adhesion^9^ and their increased motility, migration, and invasiveness^10^. Moreover, interaction with other overexpressed receptor tyrosine kinases (such as EGFR^11^, PDGFR^12^, ERBB^13^, FGFR^12^, and HGF^14^) promotes the disruption of the closed conformation and exacerbates the activation of c-Src; such a condition is widely observed in glioblastoma^15^, breast^16^, lung^17^, thyroid^18^, bones^19^^,^^20^, colon^21^, pancreas^22^, and prostate cancers^23^.

**Figure 2.**
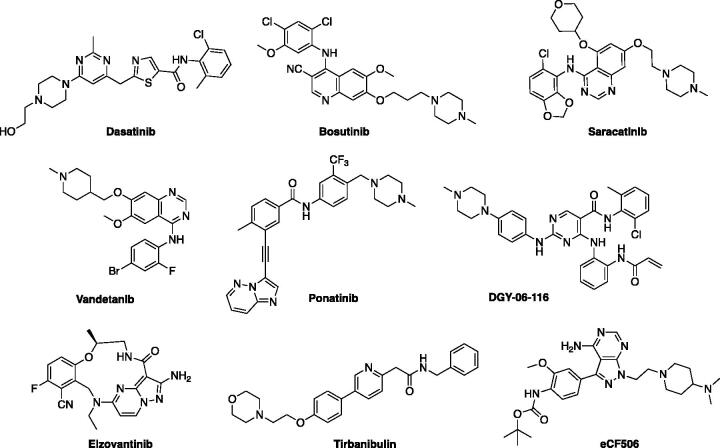
Structures of the molecules approved by the FDA or in clinical trials as Src-family inhibitors.

In this context, many efforts have been made to design and discover new molecules acting as c-Src inhibitors, blocking its downstream pathways and impairing tumour progression^24–27^. To date, five molecules targeting Src have been approved by FDA (Figure 2), and several others are in clinical trials for the treatment of solid tumours and leukemias, often in association with other cytotoxic agents. In particular, Dasatinib and Bosutinib have been approved for the treatment of chronic myeloid leukaemia (CML) in adult and paediatric patients^28^^,^^29^. Saracatinib, characterised by a more selective profile in terms of Src family inhibition^30^, is in clinical trials for the treatment of several solid tumours^24^. Vandetanib and Ponatinib are multi-tyrosine kinase inhibitors, approved by FDA to treat CML, thyroid carcinoma, and Philadelphia chromosome-positive acute lymphoblastic leukaemia in adults^31^^,^^32^. Other promising c-Src inhibitors (in phase I or II, (Figure 2) are DGY-06–116^33^, eCF506^34^, Elzovantinib (TPX-0022)^35^, and Tirbanibulin, the latter targeting the Src substrate binding site^36^ and recently approved for the topical treatment of actinic keratosis^37^.

As part of an ongoing research program aimed at identifying new chemical entities for anticancer chemotherapy, we focussed our efforts on the search of c-Src inhibitors. For this purpose, we initially screened an *in-house* collection library of small molecules characterised by great structural diversity. Analogues of the most promising compounds, containing the indolinone scaffold, were synthesised to highlight the structural determinants for the activity. Molecular modelling studies were also performed to propose a binding mode within the c-Src binding site.

## 2. Experimental

### 2.1. Computational details

Docking studies were performed on the three-dimensional structure of the complex between c-Src and **AP23464** (entry 2BDJ of the protein data bank, 2.5 Å resolution). The structure of c-Src was prepared using the Protein Preparation Wizard of the Maestro Software Package (Schrödinger suite, version 19.2). The software corrects bond orders and partial charges of the ligands and adds hydrogens to all atoms. Water molecules were removed, except those useful for stable binding interactions with the protein or ligand. Prime software was applied to define the correct conformation of missed side chains. Pre-processed protein was optimised with PROPKA and then minimised with an OPSL3 force field (convergence of heavy atoms set to a rmsd 0.3 Å).

The 2 D structures of all synthesised compounds and native ligand **AP23464** were designed using the sketcher module of the Maestro suite and prepared with the Ligprep tool, which used the Epik module to find protonation and tautomerization states of compounds at pH of 7.4 ± 0.5. To optimise the three-dimensional structures of the ligands, the Minimisation tool of the Macro model package was applied, using OPLS3 force field, while the other parameters were set to default values. Glide’s receptor grid generation wizard was used to generate a three-dimensional grid with default settings. Then, flexible docking was performed with standard precision docking mode.

### 2.2. Biological assays

*N*-Terminal His6-tagged Src was purchased from Merck-Millipore (cat. 14–326). The reaction was performed according to the manufacturer’s instructions with minor modifications: 500 μM Src-peptide (KVEKIGEGTYGVVYK), 100 μM ATP, 0.00087% NP-40, 10 ng of the enzyme, 10% DMSO in 10 μL at 30 °C for 10 min. To avoid peptide adsorption to the plastic surface, protein low-binding tubes were used. ADP-Glo Kinase Assay (Promega) was then used to detect kinase activity accordingly with manufacturer’s instruction with minor modifications. In details, reactions were transferred to white 384 well-plates and stopped by adding 10 µL of ADP-Glo Reagent (Promega) for 50 min at rt. 20 μL of Detection Reagent (Promega) was then added for 30 min and the reaction read using GloMax Discover microplate reader (Promega). Data were plotted using GraphPad Prism 5.0. ID_50_ values were obtained according to Equation (1)

(1) $$v=V/\{1+({I/\mathrm{ID}}_{50})\}$$

where v is the measured reaction velocity, V is the apparent maximal velocity in the absence of an inhibitor, I is the inhibitor concentration, and ID_50_ is the 50% inhibitory dose. Compounds tested were assumed to act as fully ATP-competitive inhibitors. Therefore, *K*_i_ values were calculated accordingly to Equation (2).

(2) $$K_{i}={\mathrm{ID}}_{50}/(1+K_{m}/[S])$$

where *K*_i_ is the affinity of the inhibitor to the enzyme, [S] is the substrate (namely, ATP) concentration, and *K*_m_ is the affinity of ATP calculated accordingly to Michaelis-Menten equation.

Cytotoxicity experiments were carried out using the human MCF7 breast cancer cell line (ATCC HTB-22). Cells were cultured in RPMI1640 plus 10% foetal bovine serum at 37 °C and 5% CO_2_. Compound potency was determined by a growth inhibition assay (CellTiter 96^®^ Aqueous One Solution Cell Proliferation Assay MTS, Promega). 24 h after seeding in a 96-well plate, cells were exposed to the compounds (concentration range 1–100 µM) and 72 h later, 20 µL of 3–(4,5-dimethylthiazol-2-yl)-5–(3-carboxymethoxyphenyl)-2–(4-sulfophenyl)-2*H*-tetrazolium salt (MTS) was added to each well. The absorbance was measured using a FLUOstar OPTIMA plate reader (BMG Labtech GmbH, Offenburg, Germany) at 492 nm after 4 h of incubation at 37 °C in 5% CO_2_. The IC_50_ is the drug concentration causing 50% cell growth inhibition determined by the dose-response curves. Experiments were performed in triplicate.

### 2.3. Chemistry

All reagents and solvents were reagent grade or were purified by standard methods before use. Melting points were determined in open capillaries by an SMP3 apparatus and are uncorrected. ^1^H and ^13 ^C NMR spectra were recorded on Bruker AV600 spectrophotometer at 600 and 150 MHz, respectively. Chemical shifts are reported in ppm (δ) relative to TMS. The coupling constants, J are reported in Hertz (Hz). All compounds were routinely checked by thin layer chromatography (TLC) using precoated silica gel 60 F_254_, aluminium foil and the spots were detected under UV light at 254 nm and 365 nm or were revealed by spraying with 10% phosphomolybdic acid in ethanol.

Compounds **1**, **2**, **4**^38^, **13**, **14**^39^, **15**, **16**^40^, **7**, **8**, **23**, **24**^41^, **9**^42^, **10**, **11**^43^, **17**^44^, **18**^45^, **19**^45^, **25**^46^, **28**^47^ are part of an in-house library. The synthetic procedures relative to the above-mentioned products have been already described and reported in the cited references.

### 2.4. General procedure for preparation of compounds 22, 27, 29, 30, 32–38, 43–50

To a solution of the benzaldehyde (0.9 mmol) in EtOH (9 ml), indolinone (100 mg, 0.6 mmol) was added, followed by piperidine (0.06 mmol). After heating at reflux for 4 h, the crude was concentrated under reduced pressure and purified by flash chromatography.

**1-hydroxy-3–(3-phenoxybenzylidene)indolin-2-one** (**22**) was prepared from 1-hydroxy-3H-indol-2-one^41^ and 3-phenoxybenzaldehyde. Purification by column chromatography (CH_2_Cl_2_/CH_3_OH 97:3) gave the title compound in 85% yield, as a yellow oil. ^1^H NMR (600 MHz, DMSO-*d*_6_, major isomer (*E*), δ: 10.90 (1H, s); 7.67 (1H, s); 7.57–7.53–7.73 (1H, m); 7.48–7.41 (2H, m); 7.40–7.37 (1H, m); 7.33–7.7.28 (1H, m); 7.26 (1H, s); 7.21–7.15 (2H, m); 7.14–7.11 (2H, m); 6.95 (1H, d, *J* = 7.6 Hz); 6.88–6.82 (1H, m). ^13 ^C NMR (150 MHz, DMSO-*d_6_*), major isomer (*E*), δ: 162.9, 157.3, 156.1, 142.4, 136.0, 135.9, 130.6, 130.4, 130.2 (× 2 C), 130.1, 124.6, 124.1, 122.1, 121.8, 119.9, 119.4 (× 2 C), 118.6, 118.3, 107.5.

**3-((5-phenylthiophen-2-yl)methylene)indolin-2-one** (**27**) was prepared from commercially available 2-oxindole and 5-phenylthiophene-2-carbaldehyde. Purification by column chromatography (CH_2_Cl_2_/CH_3_OH 95:5) gave the title compound in 95% yield, as an orange solid (m.p. 257–258 °C). ^1^H NMR (600 MHz, DMSO-*d*_6_), major isomer (*E*), δ: 10.63 (1H, s); 8.06 (1H, s); 7.90 (1H, d, *J* = 3.8 Hz); 7.79–7.73 (2H, m); 7.68 (1H, d, *J* = 7.7 Hz); 7.64 (1H, d, *J* = 3.8 Hz); 7.50–7.43 (2H, m); 7.41–7.35 (1H, m); 7.24–7.16 (1H, m); 7.02–6.97 (1H, m); 6.86 (1H, d, *J* = 7.6 Hz). ^13 ^C NMR (150 MHz, DMSO-*d_6_*), major isomer (*E*), δ: 167.4, 150.2, 140.5, 139.0, 136.9, 133.5, 129.3 (× 2 C), 128.6, 128.5.128.0, 125.8 (× 2 C), 124.4, 124.2, 121.6, 121.0, 119.4, 109.5.

**3–(3,5-dibromo-4-hydroxybenzylidene)-5,6-dimethoxyindolin-2-one** (**29**) was prepared from commercially available 5,6-dimethoxy-2-oxindole and 3,5-dibromo-4-hydroxybenzaldehyde. Purification by column chromatography (CH_2_Cl_2_/CH_3_OH 95:5) gave the title compound in 52% yield, as a yellow solid (291–292 °C). ^1^H NMR (600 MHz, DMSO-*d*_6_), *E*/Z 76/24, δ: 10.52 (1H, bs); 10.36 (1H isomer *Z*, bs); 10.32 (1H isomer *E*, bs); 8.75–8.69 (2H isomer *Z*, m); 7.95–7.89 (2H isomer *E*, m); 7.52 (1H isomer *Z*, s); 7.34 (1H isomer *Z*, s); 7.28 (1H isomer *E*, s); 7.17 (1H isomer *E*, s); 6.51 (1H isomer *E*, s); 6.45 (1H isomer *Z*, s); 3.79 (3H isomer *E*, s); 3.77 (3H isomer *Z*, s); 3.76 (3H isomer *Z*, s); 3.64 (3H isomer *E*, s). ^13 ^C-NMR (150 MHz, DMSO-*d_6_*), isomer *E* +* Z*, δ: 169.1, 167.8, 151.2 (× 2 C), 144.2 (× 2 C), 143.2 (× 2 C), 139.3, 135.6 (× 2 C), 133.1 (× 4 C), 131.5, 129.6, 128.5, 116.0, 111.8 (× 2 C), 111.4, 111.3, 107.0, 105.1, 95.5 (× 2 C), 95.0 (× 2 C), 56.3 (× 2 C), 55.7 (× 2 C).

**3-([1,1′-biphenyl]-4-ylmethylene)-5,6-dimethoxyindolin-2-one** (**30**) was prepared from 5,6-dimethoxy-2-oxindole and (1,1′-biphenyl)-4-carbaldehyde. Purification by column chromatography (CH_2_Cl_2_/CH_3_OH 95:5) gave the title compound an 83% yield, as a yellow solid (m.p. 231–232 °C). ^1^H NMR (600 MHz, DMSO-*d*_6_), major isomer (*E*), δ: 10.36 (1H, bs); 7.87–7.78 (4H, m); 7.78–7.72 (2H, m); 7.54–7.45 (4H, m); 7.43–7.36 (1H, m); 7.23 (1H, s); 3.79 (3H, s); 3.58 (3H, s). ^13 ^C NMR (150 MHz, DMSO-*d_6_*), major isomer (*E*), δ: 169.3, 151.4, 143.2, 140.9, 139.3, 138.3, 133.8, 131.8, 130.0 (× 2 C), 129.0 (× 2 C), 127.9 (× 2 C),126.8 (× 2 C), 126.7 (× 2 C), 111.7, 107.9, 95.5.

**4-((6-chloro-2-oxoindolin-3-ylidene)methyl)benzonitrile** (**32**) was prepared from **31** and 4-cyanobenzaldehyde. Purification by column chromatography (hexane/EtOAc 90:10 → 20:80) gave the title compound in 45% yield, as a yellow solid (m.p. 310 °C, dec.). ^1^H NMR (600 MHz, DMSO-*d*_6_), *E/Z* 73/27, δ:10.88 (1H isomer *Z*, s); 10.86 (1H isomer *E*, s); 8.48–8.42 (2H isomer *Z*, m); 8.04–7.98 (2H isomer *E*, m); 7.96–7.92 (3H isomer *Z*); 7.91–7.86 (2H isomer *E*, m); 7.80–7.75 (1H isomer *Z*, d, *J* = 8.3 Hz); 7.70 (1H isomer *E*, s); 7.37 (1H isomer *E*, d, *J* = 8.9 Hz); 7.10 (1H isomer *Z*, dd, *J* = 8.3 Hz, 1.8 Hz); 6.96–6.90 (2H isomer *E*, m); 6.87 (1 H isomer *Z*, d, *J* = 1.8 Hz). ^13 ^C NMR (150 MHz, DMSO-*d_6_*), major isomer (*E*), δ: 168.2, 144.7, 139.2, 134.8, 134.3, 132.7 (× 2 C), 132.0, 130.0 (× 2 C), 128.6, 124.0, 121.1, 119.3, 118.5, 110.3.

**6-chloro-3–(4-nitrobenzylidene)indolin-2-one** (**33**) was prepared from **31** and 4-nitrobenzaldehyde. Purification by column chromatography (CH_2_Cl_2_ + 0.1% Et_3_N, CH_2_Cl_2_/CH_3_OH 99:1 → 80:20, EtOAc/CH_3_OH 80:20) gave the title compound in 45% yield, as a red solid (m.p. 322–323 °C, dec.). ^1^H NMR (600 MHz, DMSO-*d*_6_), major isomer (*E*), δ: 10.89 (1H, s); 8.40–8.33 (2H, m); 8.00–7.92 (2H, m); 7.74 (1H, s); 7.40 (1H, d, *J* = 8.2 Hz); 6.95–6.90 (2H, m). ^13 ^C NMR (150 MHz, DMSO-*d_6_*), major isomer (*E*), δ: 168.1, 147.6, 144.8, 141.1, 135.0, 133.8, 130.5 (× 2 C), 129.0, 124.0 (× 2 C), 123.2, 121.2, 119.2, 110.3.

**6-chloro-3–(4-(dimethylamino)benzylidene)indolin-2-one** (**34**) was prepared from **31** and 4-(dimethylamino)benzaldehyde. Purification by column chromatography (petroleum ether/EtOAc 95:5 → 20:80, CH_2_Cl_2_/CH_3_OH 95:5) gave the title compound an 84% yield, as an orange solid (m.p. 253–254 °C). ^1^H NMR (600 MHz, DMSO-*d*_6_), major isomer (*E*), δ: 10.62 (bs,1H); 7.78 (1H d, *J* = 8.3 Hz); 7.68–7.64 (2H, m); 7.57 (1H, s); 6.96 (1H, dd, *J* = 8.3 Hz, 1.6 Hz); 6.88 (1H, d, *J* = 1.6 Hz); 6.86–6.82 (2H, m); 3.02 (6H, s). ^13 ^C NMR (150 MHz, DMSO-*d_6_*), δ: 169.8, 152.0, 143.8, 138.7, 139.9, 132.6 (× 2 C), 123.2, 121.3 (× 2 C), 121.2, 121.0, 112.1 (× 2 C), 110.1, 40.1 (× 2 C).

**6-chloro-3–(4-hydroxybenzylidene)indolin-2-one** (**35**) was prepared from **31** and 4-hydroxybenzaldehyde. Purification by column chromatography (hexane/EtOAc 90:10, CH_2_Cl_2_/CH_3_OH 95:5 → 90:10) gave the title compound in 94% yield, as a yellow solid (m.p. 330–331 °C, dec.). ^1^H NMR (600 MHz, DMSO-*d*_6_), major isomer (*E*), δ: 10.70 (1H, bs); 10.27 (1H, bs); 7.69 (1H, d, *J* = 8.2 Hz); 7.66–7.60 (2H, m); 7.59 (1H, s); 6.97–6.91 (3H, m); 6.90–6.99 (1H, m). ^13 ^C NMR (150 MHz, DMSO-*d_6_*), isomer *E* +* Z*, δ: 169.0, 167.3, 160.5, 159.6, 143.8, 141.1, 138.5, 137.5, 135.0, 133.3, 132.0 (× 4 C), 125.4, 124.7, 124.6, 123.4, 123.2, 121.6, 120.7, 120.5, 120.4, 120.3, 115.7 (× 2 C), 115.3 (× 2 C), 109.8, 109.0.

**4-((6-chloro-2-oxoindolin-3-ylidene)methyl)benzoic acid** (**36**) was prepared from **31** and 4-formylbenzoic acid. Purification by column chromatography (hexane/EtOAc 90:10 → 75:25, CH_2_Cl_2_/CH_3_OH 95:5 → 80:20, EtOAc/CH_3_OH 80:20) gave the title compound in 22% yield, as a yellow solid (m.p. 271–272 °C, dec.). ^1^H NMR (600 MHz, DMSO-*d*_6_), major isomer (*E*), δ: 10.86 (1H, bs); 8.10–8.03 (2H, m); 7.81–7.73 (2H, m); 7.71 (1H, s); 7.48 (1H, d, *J* = 7.48 Hz); 6.96–6.89 (2H, m). ^13 ^C NMR (150 MHz, DMSO-*d_6_*), major isomer (*E*), δ: 168.4, 168.0, 144.5, 137.5, 135.6, 134.4, 131.6, 129.7 (× 2 C), 129.1 (× 2 C), 127.5, 123.8, 121.0, 119.6, 110.2.

**3–(4-(benzyloxy)benzylidene)-6-chloroindolin-2-one** (**37**) was prepared from **31** and 4-benzyloxybenzaldehyde. Purification by column chromatography (hexane/EtOAc 90:10 → 75:25) gave the title compound in 50% yield, as a yellow solid (m.p. 239–240 °C, dec.). ^1^H NMR (600 MHz, DMSO-*d_6_*), *E/Z* 75/25, δ:10.74 (1H isomer *E* + 1H isomer *Z*, s); 8.48 (2H isomer *Z*, m); 7.82 (1H isomer *Z*, s); 7.78–7.69 (2H isomer *E* + 1H isomer *Z*, m); 7.68– 7.61 (2H, m); 7.54–7.48 (2H isomer *E*, m); 7.47–7.40 (2H isomer *E* + 2H isomer *Z*, m); 7.39–7.34 (2H isomer *Z*, m); 7.22–7.16 (2H isomer *E*, m); 7.16–7.11 (2H isomer *Z*, m); 7.07–7.02 (1H isomer *Z*, m); 6.95 (1H isomer *E*, dd, *J* = 8.4 Hz, 2.1 Hz); 6.90 (1H isomer *E*, d, *J* = 2.1 Hz); 6.86–6.83 (1H isomer *Z*, m); 5.21 (2H isomer *E* + 2H isomer *Z*, s). ^13 ^C NMR (150 MHz, DMSO-*d_6_*), isomer *E* +* Z*, δ: 168.8, 167.2, 160.51, 59.8, 144.0, 141.3, 137.9, 136.9, 136.6, 134.6, 133.6, 132.2, 131.7 (× 2 C), 128.5 (× 6 C), 128.0 (× 2 C), 127.8 (× 4 C), 127.0, 126.6, 124.5, 124.3, 123.3, 122.9, 120.8, 120.7 (× 2 C), 120.1, 115.1 (× 2 C), 114.6 (× 2 C), 110.0, 109.1.

**6-chloro-3–(3-phenoxybenzylidene)indolin-2-one** (**38**) was prepared from **31** and 3-phenoxybenzaldehyde. Purification by column chromatography (hexane/EtOAc 90:10 + 0.1% Et_3_N, hexane/EtOAc 80:20 → 50:50, CH_2_Cl_2_/CH_3_OH 99:1 → 80:20) gave the title compound in 19% yield, as a yellow solid (m.p. 188–189 °C). ^1^H NMR (600 MHz, DMSO-*d*_6_), major isomer (*E*), δ: 10.78 (1H, bs); 7.63 (1H, s); 7.58–7.52 (1H, m); 7.49–7.41 (3H, m); 7.40–7.35 (1H, m); 7.25–7.19 (2H, m); 7.18–7.10 (3H, m); 6.91–6.82 (1H, m). ^13 ^C NMR (150 MHz, DMSO-*d_6_*), major isomer (*E*), δ: 168.4, 157.3, 156.0, 144.4, 136.0, 135.7, 134.2, 130.6, 130.2 (× 2 C), 126.9, 124.4, 124.1, 123.7, 120.8, 119.8, 119.5 (× 2 C), 119.4, 118.2, 110.1.

#### 2.4.1. Ethyl 2–(4-((6-chloro-2-oxoindolin-3-ylidene)methyl)phenoxy) acetate (43)

To a solution of 4-hydroxybenzaldehyde (100 mg, 0.82 mmol) and bromoethyl acetate (0.14 ml, 1.23 mmol) in dry acetone (3 ml), K_2_CO_3_ (170 mg, 1.64 mmol) was added, and the reaction was left stirring at reflux. After 4 h, the crude was concentrated under reduced pressure, treated with EtOAc and washed with water. The resulting organic phase was washed with brine and dried over Na_2_SO_4_, then evaporated under reduced pressure. Purification by flash chromatography (petroleum ether/EtOAc 90:10 → 85:15) afforded ethyl 2–(4-formylphenoxy)acetate **40** (150 mg, 88% yield) as a yellow oil. ^1^H NMR (600 MHz, DMSO-*d*_6_), δ: 9.93 (1H, s); 7.91–7.85 (2H, m); 7.07–7.02 (2H, m); 4.73 (2H, s); 4.31 (2H, q, *J* = 6.6 Hz); 1.33 (3H, t, *J* = 6.6 Hz).

Compound **43** was prepared from **31** and the above compound. Purification by column chromatography (hexane/EtOAc 90:10 → 75:25) gave the title compound in 64% yield, as a yellow solid (m.p. 158–159 °C). ^1^H NMR (600 MHz, DMSO-*d*_6_), *E*/*Z* 90/10, δ: 10.74 (1H isomer *E*, bs); 8.49–8.44 (2H isomer *Z*, m); 7.82 (1H isomer *Z*, s); 7.75–7.68 (2H isomer *E*, m); 7.76–7.58 (2H isomer *E*, m); 7.13–7.07 (2H isomer *E*, m); 7.06–7.03 (1H isomer *Z*, m); 6.94 (1H isomer *E*, dd, *J* = 1.9 Hz, 8.4 Hz); 6.90 (1H isomer *E*, d, J = 1.9 Hz); 6.84 (1H isomer *Z*, d, *J* = 1.8 Hz); 4.90 (2H isomer *E* + 2H isomer *Z*, s); 4.21 (2H isomer *E* + 2H isomer *Z*, q, *J* = 6.9 Hz); 1.24 (3H isomer *E* + 3H isomer *Z*, t, *J* = 6.9 Hz). ^13 ^C NMR (150 MHz, DMSO-*d_6_*), isomer *E* +* Z*, δ: 168.8, 168.4 (× 2 C), 167.2, 159.6, 159.0, 144.0, (× 2 C), 141.4, 137.7, 136.7 (× 2 C), 134.4 (× 2 C), 133.7, 132.3, 131.5 (× 2 C), 127.4, 127.0, 124.8, 124.3, 123.3 (× 2 C), 120.8 (× 3 C), 120.0, 114.9 (× 2 C), 114.3 (× 2 C), 64.7, 64.6, 60.7 (× 2 C), 14.0 (× 2 C).

#### 2.4.2. Methyl-4-((4-((6-chloro-2-oxoindolin-3-ylidene)methyl) phenoxy)methyl)benzoate (44)

To a solution of 4-hydroxybenzaldehyde (100 mg, 0.82 mmol) and methyl-4-bromomethyl benzoate (205 mg, 0.9 mmol) in dry DMF (3 ml), Cs_2_CO_3_ (200 mg, 0.57 mmol) was added, and the reaction was left stirring at rt. After 16 h, the crude was concentrated under reduced pressure, treated with EtOAc and washed with water. The resulting organic phase was washed with brine and dried over Na_2_SO_4_, then evaporated under reduced pressure. Purification by flash chromatography (petroleum ether/EtOAc 90:10 → 75:25) afforded methyl 4-((4-formylphenoxy)methyl)benzoate **41** (175 mg, 79% yield) as a white solid. ^1^H NMR (600 MHz, DMSO-*d*_6_), δ: 9.93 (1H, s); 8.14–8.08 (2H, m); 7.91–7.84 (2H, m); 7.56–7.50 (2H, m); 7.14–7.07 (2H, m); 5.24 (2H, s); 3.95 (3H, s).

Compound **44** was prepared from **31** and the above compound. Purification by column chromatography (hexane/EtOAc 90:10 → 50:50) gave the title compound in 93% yield, as a yellow solid (m.p. 201–202 °C). ^1^H NMR (600 MHz, DMSO-*d*_6_), *E*/*Z* 70/30, δ: 10.73 (1H isomer *Z*, bs); 10.71 (1H isomer *E*, bs); 8.49–8.43 (2H, isomer *Z*, m); 8.05–7.96 (2H isomer *E*, m); 7.79 (1H isomer *Z*, s); 7.74–7.66 (2H isomer *E* + 2H isomer *Z*, m); 7.65–7.58 (4H isomer *E* + 3H isomer *Z*, m); 7.19–7.15 (2H isomer *E*, m); 7.14–7.11 (2H isomer *Z*, m); 7.02 (1H isomer *Z*, dd, *J* = 1.6 Hz, 8.2 Hz); 6.92 (1H isomer *E*, dd, *J* = 1.6 Hz, 8.3 Hz); 6.87 (1H isomer *E*, d, *J* = 1.6 Hz); 6.81 (1H isomer z, d, *J* = 1.6 Hz); 5.29 (2H isomer *E* + 2H isomer *Z*, s); 3.85 (3H isomer *E* + 2H isomer *Z*, s). ^13 ^C NMR (150 MHz, DMSO-*d_6_*), isomer *E* +* Z*, δ: 168.8, 167.2, 166.0 (× 2 C), 160.2, 159.6, 144.0, 142.2 (× 2 C), 141.4 137.8, 136.7, 134.6, 133.6, 131.7, 131.6 (× 2 C), 129.4 (× 6 C), 129.1 (× 2 C), 127.2 (× 4 C), 127.2, 126.8, 124.6, 124.3, 123.3, 123.1, 120.8 (× 3 C), 120.1, 115.2 (× 2 C), 114.6 (× 2 C), 110.0, 109.1, 68.8 (× 2 C), 52.1 (× 2 C).

#### 2.4.3. 6-Chloro-3–(4-(cyclopropylmethoxy)benzylidene)indolin-2-one (45)

To a solution of 4-hydroxybenzaldehyde (100 mg, 0.82 mmol) and bromomethyl cyclopropane (0.16 ml, 1.64 mmol) in dry acetone (3 ml), K_2_CO_3_ (450 mg, 3.28 mmol) was added, and the reaction was left stirring at reflux. After 16 h, the crude was concentrated under reduced pressure, treated with EtOAc and washed with water. The resulting organic phase was washed with brine and dried over Na_2_SO_4_, then evaporated under reduced pressure. Purification by flash chromatography (petroleum ether/EtOAc 95:5 → 90:10) afforded compound **42** (110 mg, 76% yield) as a colourless oil. ^1^H NMR (600 MHz, DMSO-*d*_6_), δ: 9.90 (1H, s); 7.93–7.81 (2H, m); 7.06–6.95 (2H, m); 3.92 (2H, d, *J* = 6.9 Hz); 1.37–1.26 (1H, m); 0.77–0.62 (2H, m); 0.50–0.34 (2H, m).

Compound **45** was prepared from **31** and the above compound. Purification by column chromatography (hexane/EtOAc 90:10 → 70:30) gave the title compound in 47% yield, as a yellow solid (m.p. 231–232 °C). ^1^H NMR (600 MHz, DMSO-*d*_6_), isomer *E*, δ: 10.74 (1H, bs), 8.42–8.50 (2H, m); 7.81 (1H, s); 7.71 (1H, d, *J* = 8.1 Hz); 7.10–6.99 (3H, m), 6.81 (1H, s); 3.93 (2H, d, J = 6.7 Hz); 1.32–1.22 (1H, m); 0.67–0.53 (2H, m); 0.42–0.31 (2H, m). ^13 ^C NMR (150 MHz, DMSO-*d_6_*), major isomer (*E*), δ: 167.2, 160.9, 141.3, 138.0, 134.6 (× 2 C), 132.1, 131.6, 126.7, 124.4, 122.7, 120.6, 114.2 (× 2 C), 109.1, 72.3, 10.0, 3.1 (× 2 C).

#### 2.4.4. 2–(4-((6-Chloro-2-oxoindolin-3-ylidene)methyl)phenoxy)acetic acid (46)

To a solution of compound **43** (60 mg, 0.17 mmol) in THF/water 2:1 (6 ml), LiOH monohydrate (18 mg, 0.42 mmol) was added and the reaction was left stirring at rt. After 16 h, the mixture was concentrated under reduced pressure and extracted twice with Et_2_O. The resulting aqueous phase was treated with HCl 37% until pH = 2, then extracted three times with EtOAc, washed with brine, dried over Na_2_SO_4_, then evaporated under reduced pressure to afford 40 mg of **46** in 71% yield, as an orange solid (m.p. 197 °C, dec.). ^1^H NMR (600 MHz, DMSO-*d*_6_), *E*/*Z* 74/26, δ: 13.08 (1H isomer *E* + 1H isomer *Z*, bs); 10.75 (1H isomer *Z*, bs); 10.73 (1H isomer *E*, bs); 7.90–7.86 (2H isomer *Z*, m); 7.82 (1H isomer *Z*, s); 7.74–7.68 (2H, isomer *Z*, m); 7.66–7.61 (1H isomer *E* + 1H isomer *Z*, m); 7.48 (1H, d, *J* = 8.3 Hz); 7.11–7.15 (2H isomer *Z*, m); 7.10–7.06 (2H isomer *E* + 1H isomer *Z*, m); 6.97–6.93 (1H isomer *E* + 1H isomer *Z*); 6.90 (1H isomer *E*, d, *J* = 2.2 Hz); 4.84 (2H isomer *Z*, s); 4.80 (2H isomer *E*, s). ^13 ^C NMR (150 MHz, DMSO-*d_6_*), major isomer (*E*), δ: 169.9, 168.8, 159.2, 144.0, 136.7, 133.7, 131.6 (× 2 C), 126.8, 124.6, 123.3, 120.8, 120.0, 114.8, 110.0, 64.6.

**4-((4-((6-chloro-2-oxoindolin-3-ylidene)methyl)phenoxy)methyl)benzoic acid** (**47**). To a solution of compound **44** (70 mg, 0.17 mmol) in THF/water 2:1 (6 ml), LiOH monohydrate (18 mg, 0.42 mmol) was added and the reaction was left stirring at rt. After 16 h, the mixture was concentrated under reduced pressure and extracted twice with Et_2_O. The resulting aqueous phase was treated with HCl 37% until pH = 2, then extracted three times with EtOAc, washed with brine, dried over Na_2_SO_4_, then evaporated under reduced pressure to afford 60 mg of **47** in 87% yield, as a yellow solid (m.p. 279–280 °C). ^1^H NMR (600 MHz, DMSO-*d*_6_), major isomer (*E*), δ: 13.08 (1H, bs); 10.73 (1H, bs); 8.04–7.96 (2H, m); 7.77–7.71 (2H, m); 7.66–7.56 (4H, m); 7.22–7.17 (2H, m); 6.94 (1H, dd, *J* = 1.4 Hz, 8.6 Hz); 6.90 (1H, d, *J* = 1.4 Hz); 5.32 (2H, s). ^13 ^C NMR (150 MHz, DMSO-*d_6_*), major isomer (*E*), δ: 168.8, 167.0, 159.6, 144.0, 141.7, 136.8, 133.6, 131.8 (× 2 C), 130.3, 129.5 (× 2 C), 127.5 (× 2 C), 126.7, 124.6, 123.3, 120.8, 120.1, 110.0, 69.0.

**6-chloro-3-((5-phenylthiophen-2-yl)methylene)indolin-2-one** (**48**) was prepared from **31** and 5-phenylthiophene-2-carbaldehyde. Purification by column chromatography (hexane/EtOAc 90:10, CH_2_Cl_2_/CH_3_OH 95:5 → 90:10) gave the title compound 98% yield, as a red solid (m.p. 233–234 °C). ^1^H NMR (600 MHz, DMSO-*d*_6_), major isomer (*E*), δ: 10.80 (1H, bs); 8.18 (1H, s); 7.93 (1H, d, *J* = 3.4 Hz); 7.81–7.76 (2H, m); 7.72 (1H, d, *J* = 8.3 Hz); 7.68 (1H, d, J = 3.4 Hz); 7.53–7.47 (2H, m); 7.44–7.38 (1H, m); 7.07 (1H, dd, *J* = 8.3 Hz, 1.9 Hz); 6.89 (1H, d, J = 1.9 Hz). ^13 ^C NMR (150 MHz, DMSO-*d_6_*), major isomer (*E*), δ: 167.2, 150.7, 141.5, 139.5, 136.7, 133.4, 132.4, 129.3 (× 2 C), 129.0, 128.7, 125.8 (× 2 C), 124.3, 123.4, 120.8 (× 2 C), 120.3, 109.4.

**3-((2-bromothiazol-5-yl)methylene)-6-chloroindolin-2-one** (**49**) was prepared from **31** and 2-bromo-5-formylthiazole. Purification by column chromatography (petroleum ether/EtOAc 95:5 → 10:90, CH_2_Cl_2_/CH_3_OH 95:5 → 85:15) gave the title compound an 81% yield, as a red solid (m.p. 262–263 °C, dec.). ^1^H NMR (600 MHz, DMSO-*d*_6_), major isomer (*E*), δ: 10.99 (bs, 1H); 8.23 (2H, s); 7.71 (1H, d, *J* = 8.4 Hz); 7.11 (1H, dd, *J* = 8.4 Hz, 1.9 Hz); 6.92 (1H, d, J = 1.9 Hz). ^13 ^C NMR (150 MHz, DMSO-*d_6_*), major isomer (*E*), δ: 167.4, 151.2, 143.7, 142.1, 135.6, 133.5, 124.8, 123.0, 122.3, 121.5, 121.3, 109.9.

**3-((1H-indol-5-yl)methylene)-6-chloroindolin-2-one** (**50**) was prepared from **31** and 1*H*-indole-5-carbaldehyde. Purification by column chromatography (hexane/EtOAc 95:5 → 20:80, CH_2_Cl_2_:CH_3_OH 95:5 → 90:10) gave the title compound in 34% yield, as a yellow solid (m.p. 238–239 °C). ^1^H NMR (600 MHz, DMSO-*d*_6_), major isomer (*E*), δ: 11.46 (1H, bs); 10.70 (1H, s); 7.98 (1H, s); 7.82 (1H, s); 7.77 (1H, d, *J* = 8.1 Hz); 7.55 (1H, d, *J* = 8.6 Hz); 7.51 (1H, d, *J* = 8.6 Hz); 7.49–7.46 (1H, m); 6.96–6.92 (1H, m); 6.92–6.88 (1H, m). ^13 ^C NMR (150 MHz, DMSO-*d_6_*), major isomer (*E*), δ: 169.1, 152.5, 143.8, 139.6, 136.8, 133.2, 127.8, 127.0124.8, 123.4, 122.9 (× 3 C), 120.7, 120.5, 111.8, 109.8, 102.1.

## 3. Results and discussion

### 3.1. Synthesis and biological activity evaluation

A small collection of molecules containing highly diverse chemical scaffolds was initially screened on c-Src to evaluate their inhibiting activity at a 100 μM concentration (Table 1). Triazolo-triazine **13** showed the best inhibition potency (90%). However, its closely related analogue **14** did not affect the enzyme activity. Indolinone and *N*-hydroxyl indolinone derivatives **7**–**9** exhibited a significant activity (percent inhibition ranging from 77 to 84%). A modest inhibition profile was highlighted for azaindole **1** and kynurenic derivative **17** (about 58%), followed by thiazolylbenzoquinone **10** (43%) and dihydroquinolin-2-one **12** (40%), whereas all the other entries of the library showed low to no activity towards c-Src. ID_50_ of both triazolo-triazine **13** and indolinone **8** were in the two-digit micromolar concentration (50 and 12.5 µM, respectively).

**Table 1.** Evaluation of the inhibitory activity of c-Src by the *in-house* prepared collection of small molecules.

Compound	% Inhibition Src (100 μM)	ID_50_	Compound	% Inhibition Src (100 μM)	ID_50_	
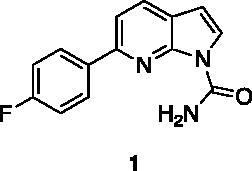	58.1%		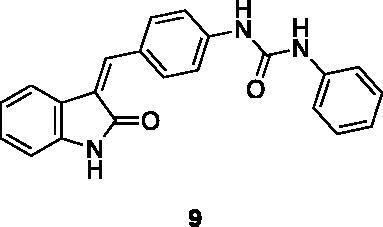	84.0%		
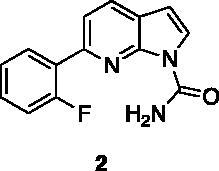	28.6%		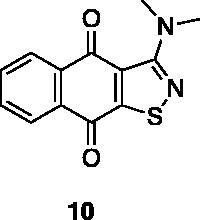	43.5		
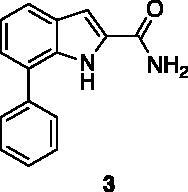	34.8%		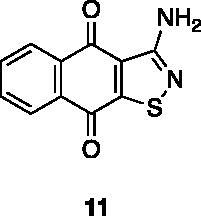	2.2%		
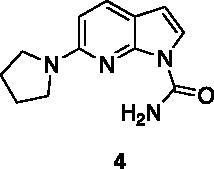	4.0%		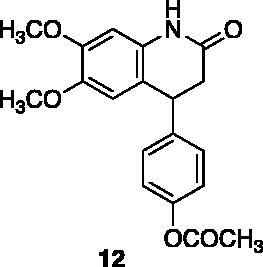	40.0%		
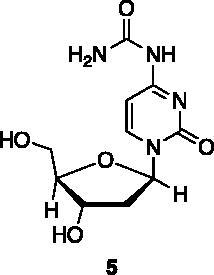	1.8%		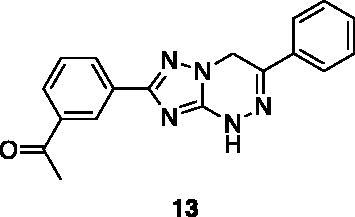	90.0%	50.0 µM	
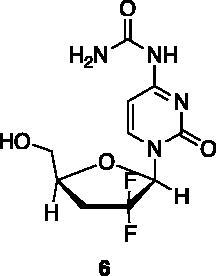	10.3%		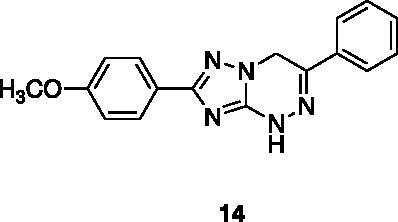	n.d.		
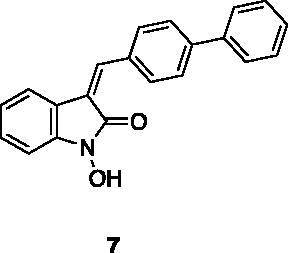	77.3%		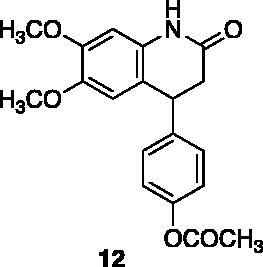	7.8%		
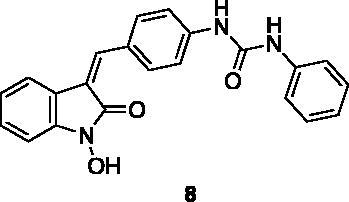	83.2%	12.5 μM	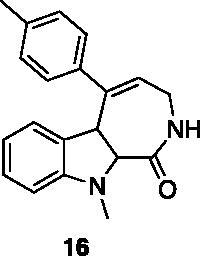	0.6%		
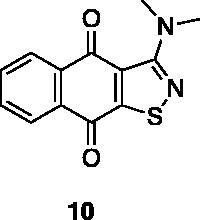	57.6%		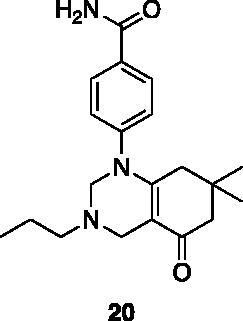	7.7%		
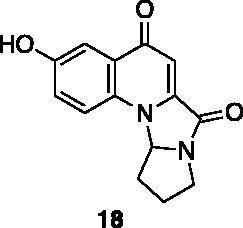	15.5%		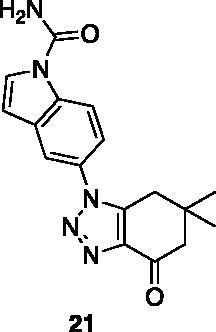	n.d.		
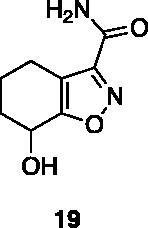	12.8%		**Dasatinib**	100.0%		1.6 nM

Dasatinib was used as reference compound.

These results prompted us to focus on the indolinone core for further investigations. This skeleton can be considered a very attractive target for biological activity evaluation and SAR studies, offering the possibility of easy chemical accessibility and several points for chemical decoration. We thus prepared and tested a second-generation set of compounds containing the indolinone scaffold substituted at positions 5 and/or 6 and bearing an arylidene side chain appended to position 3. We also prepared **24** to evaluate the role of the heteroaromatic system and both **22** and **23** to investigate the effect of the OH group on the nitrogen atom. All the compounds showed a good dose-dependent activity at 100 μM and 10 μM concentrations, with **26** having the best inhibitory potency of the series (95 and 67% at 100 and 10 μM, respectively) (Table 2).

**Table 2.** Inhibitory activity evaluation of indolinones **22–30**.

	% Inhibition			
Compound	100 µM	10 µM	ID_50_ (µM)	K*i* (ATP competitive)
**Dasatinib**	100	100	1.60 nM ± 0.22 nM	0.80 nM ± 0.11 nM
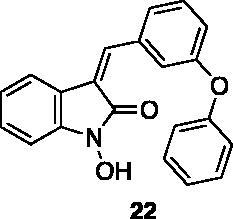	64.61 ± 3.80	32.06 ± 3.53		
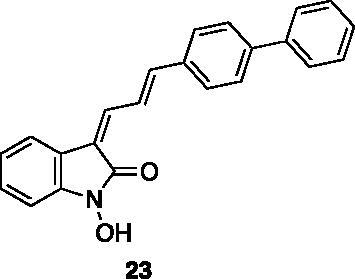	57.44 ± 6.31	22.77 ± 2.94		
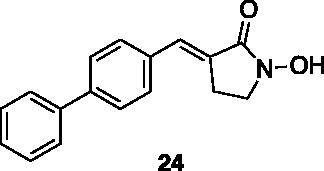	87.46 ± 7.41	34.18 ± 0.60		
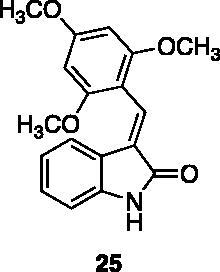	35.79 ± 3.69	23.15 ± 5.55		
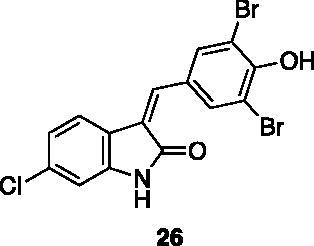	94.70 ± 0.30	67.03 ± 0.10	5.63 μM ± 2.46 μM	3.80 µM ± 0.63 μM
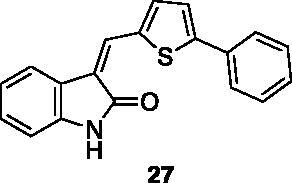	45.58 ± 7.85	25.30 ± 9.71		
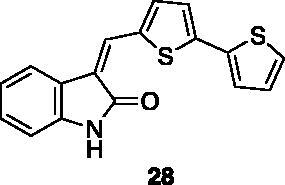	55.15 ± 0.91	30.69 ± 10.16		
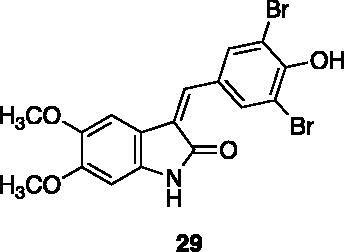	78.56 ± 1.32	39.81 ± 3.16		
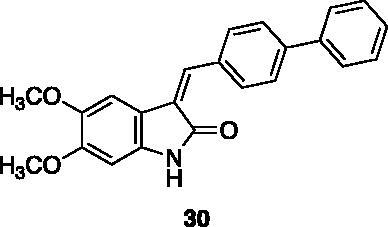	42.03 ± 8.91	9.39 ± 19.22		

Dasatinib was used as reference compound.

**Scheme 1.**
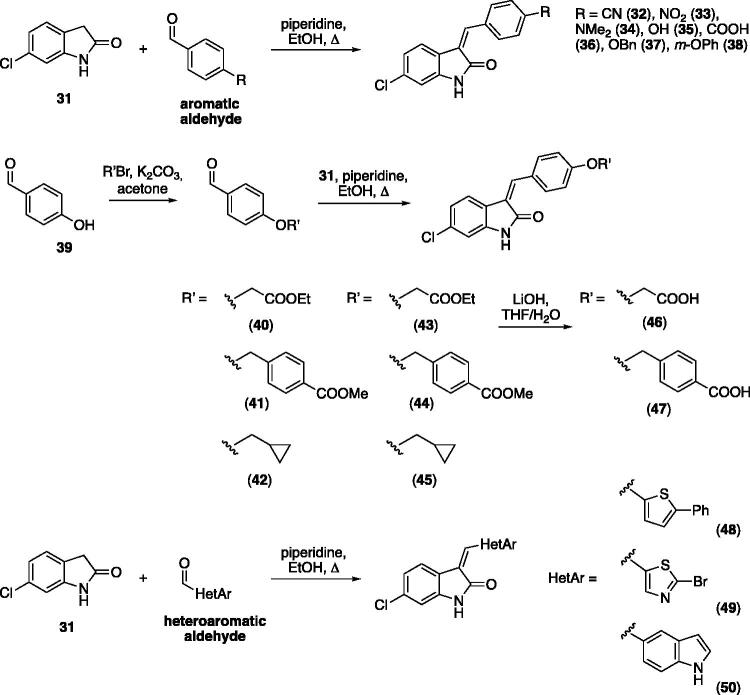
General synthesis by Knoevenagel condensation starting from 6-chloro oxindole and different aromatic and heteroaromatic compounds.

Compound **26** was prioritised and its structure was used as the starting point to prepare new analogues, maintaining unaltered the 6-chloro oxindole moiety and introducing decorations on the aromatic ring connected by a methylene bridge to C3. Knoevenagel condensation conditions were exploited to react 6-chloro oxindole **31** with substituted aromatic/heteroaromatic aldehydes (Scheme 1), obtaining 15 new variously substituted derivatives.

All the analogues contained a phenyl ring connected by a conjugated vinyl fragment to C3. Various substituents with different stereoelectronic properties were introduced in the para position of the ring: cyano (**32**), nitro (**33**), dimethylamino (**34**), phenolic (**35**) and carboxylic (**36**) groups. Commercially available *p*-benzyloxy benzaldehyde and *m*-phenoxy benzaldehyde were employed for the preparation of **37** and **38**. Additionally, alkylation of 4-hydroxy benzaldehyde **39** as in **43**–**47** gave the opportunity to increase the overall compound length and eventually fill some empty regions of the cavity, either in presence of aromatic or aliphatic moieties, as well as with a terminal acidic or ester portion. Finally, compounds containing heterocyclic substituents were synthesised (**48–50**) to investigate their potential role as hydrogen bond acceptors/donors.

All the obtained compounds were characterised by NMR analysis, showing the presence of isomeric mixtures (from 9:1 to 7:3 *E/Z* isomers). The relative ratios of the *E* and *Z* stereoisomers within this series (**32**–**50**) were assigned based on the chemical shifts of protons at the C2’ and C6’ positions^41^. Previous investigations on 3-arylideneoxindoles have reported the *E/Z* isomerisation to be solvent-, temperature-, time-, and light-dependent^48–50^. For this reason, the compounds were tested as mixtures of the two isomers.

The percentage of inhibition at the concentrations of 100 μM and 10 μM, ID_50_ of the most active compounds, and their *K*_i_ were evaluated and reported in Table 3. Compounds **32**–**34** showed a good inhibitory activity towards the enzyme, with **34** having the best profile of inhibition (85% at 10 µM). Conversely, **36**, with a carboxylic group directly linked to the phenyl ring, had very low activity. However, the percentage of inhibition was enhanced by increasing the length of the spacer between the aromatic ring and the acidic group (**46**, 70% at 100 µM, **47** 94% at 100 µM), with the corresponding esters (**43** and **44**) showing a good activity as well. Replacement of the acidic group of **46** with a hydrophobic cyclopropyl substituent led to an almost inactive compound (**45**). Moreover, the phenolic derivative **35** and the corresponding benzyl ether **37** maintained high inhibitory activity at the highest dose, which significantly decreased at 10 µM. The shift of a lipophilic aromatic ring to *meta* position on the phenyl group (**38**) did not influence the activity. Among compounds containing heterocyclic rings, **50** showed the best activity at both concentrations, whereas the thiophene- and thiazole-containing compounds **48** and **49**, respectively, evidenced a drop in activity, in particular at the lowest concentration.

**Table 3.** Evaluation of the inhibitory activity of the novel indolinone derivatives.

	% Inhibition			
Compound	100 µM	10 µM	ID_50_	*K_i_* (ATP competitive)
**Dasatinib**	100	100	1.60 nM ± 0.22 nM	0.80 nM ± 0.11 nM
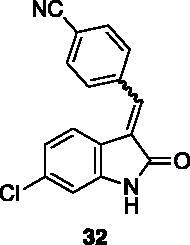	88.96 ± 1.35	35.74 ± 9.66	>10 µM	–
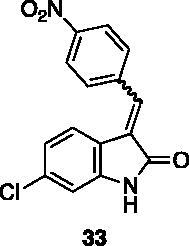	69.94 ± 0.19	53.03 ± 18.08	2.43 µM ± 1.03 µM	1.64 µM ± 0.69 µM
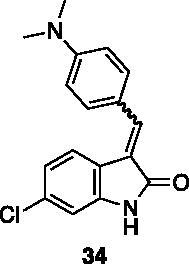	75.72 ± 7.63	85.35 ± 6.98	0.71 µM ± 0.43 µM	0.48 µM ± 0.29 µM
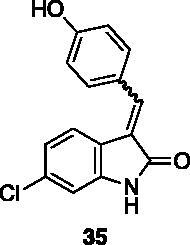	86.86 ± 2.50	37.90 ± 0.35	>10 µM	–
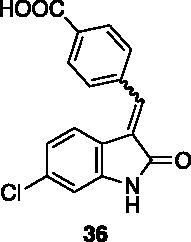	47.19 ± 9.33	4.15 ± 1.28	–	–
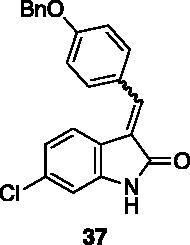	84.27 ± 0.82	16.86 ± 13.95	–	–
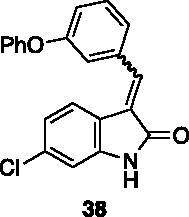	53.62 ± 12.91	31.48 ± 3.16	–	–
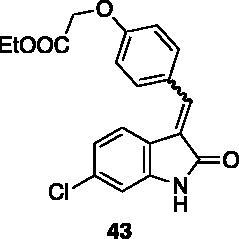	80.84 ± 6.47	71.67 ± 2.27	3.24 µM ± 0.43 µM	2.19 µM ± 1.03 µM
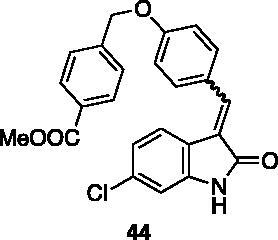	92.13 ± 3.84	41.05 ± 10.30	–	–
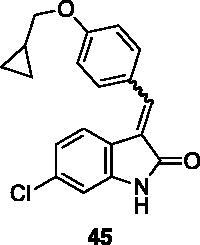	34.40 ± 13.26	14.44 ± 2.09	–	–
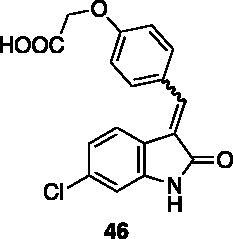	70.56 ± 4.80	0	–	–
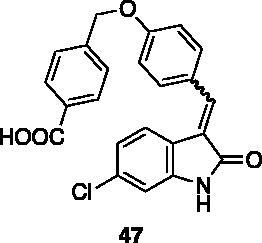	93.88 ± 0.58	63.39 ± 1.06	5.31 µM ± 0.47 µM	3.58 µM
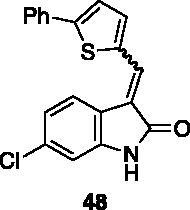	87.71 ± 3.04	12.83 ± 1.32	–	–
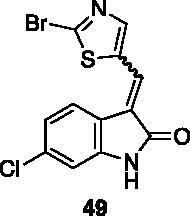	76.82 ± 9.22	53.85 ± 26.57	>10 µM	–
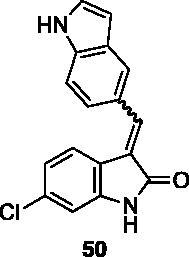	96.08 ± 1.42	41.20 ± 1.12	9.14 µM ± 1.74 µM	6.17 µM

Dasatinib was used as reference compound.

Compound **34** resulted to be the best of the series, showing significant inhibition of the enzyme at 10 µM (85%), with ID_50_ and *K*_i_ in the sub-micromolar range (0.71 and 0.48 µM, respectively). These findings suggest that the presence of an anilino nitrogen could have an important role in the inhibitory activity of the compounds.

With these results in our hands, the cytotoxicity test of selected molecules was carried out on the MCF-7 breast cancer cell line and compared to the c-Src inhibitor Dasatinib used as standard (Table 4). Thiazole **49** showed potency comparable to Dasatinib. Ester **43** and indole derivative **50** were able to exert some relevant but not excellent cytotoxic activity. It is worth noting that **34**, the best inhibitor in the cell-free assay, did not show any cytotoxic activity, probably due to pharmacokinetic issues.

**Table 4.** Cytotoxicity evaluation of selected compounds on human MCF-7 breast cancer cell line.

Compound	MCF-7 (IC_50_, μM)
**Dasatinib**	27 ± 1
**33**	>100
**34**	>100
**43**	85 ± 2
**49**	33 ± 2
**50**	68 ± 2

24 h after seeding, cells were exposed for 72 h to the compounds and cytotoxicity was measured using MTS assay. Experiments were performed in triplicate and data represent mean values ± SD. Dasatinib was used as reference compound.

### 3.2. Molecular docking studies

**Figure 3.**
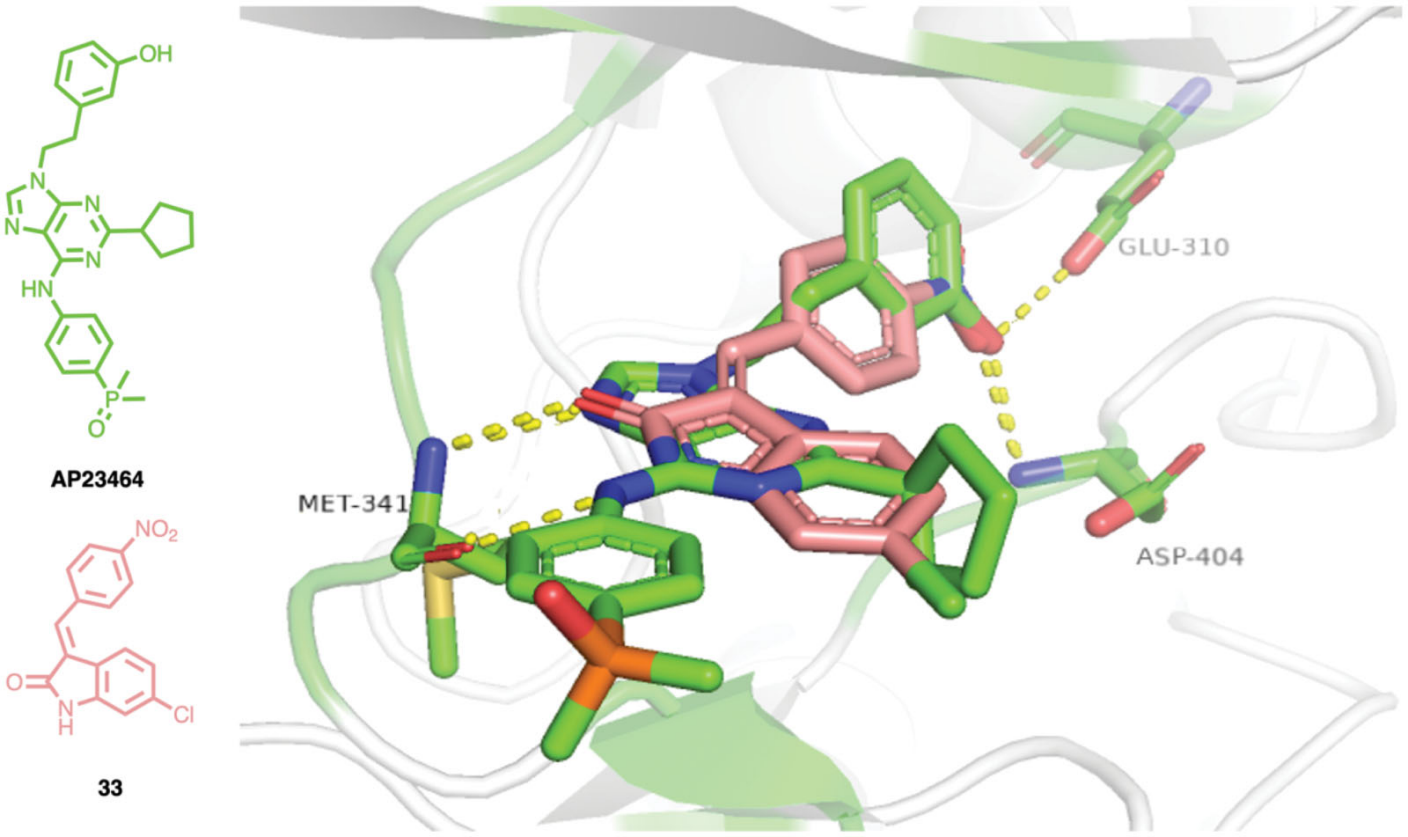
Graphical representation of the best-scored binding pose of **33** (magenta) within the c-Src ATP binding site, in comparison to the co-crystallized inhibitor **AP23464** (green). Yellow dashed lines represent hydrogen bonds between Met341, Glu310, Asp404, and both ligands.

In the attempt to find a putative binding mode of the new indolinone derivatives within the ATP-binding site of c-Src, molecular docking simulations were performed using the software Glide. For this purpose, the three-dimensional structure of the co-crystallized complex between c-Src and **AP23464** (entry 2BDJ of the protein data bank, 2.50 Å resolution) was used for calculations. Preliminary validation of the docking protocol was done by re-docking **AP23464** into the binding site. The resulting rmsd = 0.903 Å, which was calculated on the atomic coordinates of the inhibitor, confirmed the reliability of the docking protocol. Two highly conserved hydrogen bonds were found between N7 and aniline nitrogen of **AP23464** with the amide nitrogen and the carbonyl of the backbone of the Met341, respectively (Figure 3). The 3-hydroxyphenylethyl group in position N9 on the purine scaffold protruded into the hydrophobic pocket and mediated a series of interactions. Specifically, the hydroxyl substituent formed hydrogen bonds with the carboxyl group of the Glu310 side chain and the backbone NH group of Asp404.

Considering that the new compounds were assayed as *E*/*Z* isomers, isomerisation was allowed during calculations. Docking scores showed that for each compound, the *E*-isomer was preferred to the *Z*-isomer. Overall, the indolinone derivatives showed a common binding pose with the lactam moiety that mimicked the hydrogen bond acceptor-donor motif represented by the N9 and the anilino NH group of **AP23464**. They served as hydrogen bond acceptor-donor system to give the classical interactions with Met341 of the hinge region (Figure 3). Moreover, the chlorophenyl moiety was superimposable to the cyclopentyl ring of **AP23464**. Finally, the aryl appendage at C3 could mimic the phenol portion of the co-crystallized inhibitor. As an example, the predicted binding mode of **33**, which showed single-digit micromolar ID_50_ and *K*_i_, was able to maintain the two hydrogen bonds with Met341 in its best-scored pose (Figure 3). In addition, one of the oxygen atoms of the nitro group matched the phenol oxygen of **AP23464**, thus mimicking its ability to make an additional hydrogen bond with Asp404. A very similar binding mode was found for **50**, where the NH group of the pendant indole nucleus was able to build a hydrogen bond with the side chain of Glu310, in addition to the classical interactions with Met341 (Figure S1). On the other hand, molecular docking and scoring were not able to justify the binding affinity of **34**, whose peripheral dimethylanilino moiety gave hydrophobic interactions with Ala403, whereas other interactions were not highlighted, in addition to the classical contacts with Met341 (Figure S2).

Increasing the length and bulkiness of the substituent at the pendant phenyl ring forced the terminal molecular edge outside the hydrophobic side pocket where the phenol ring of **AP23464** was accommodated. As an example, the benzoic carboxyl group of the long side chain of **47**, which retained the two hydrogen bonds with Met341, made two additional hydrogen bonds with Cys277 and Phe278. On the contrary, the docking pose of the corresponding methyl ester **44** lacked the fundamental anchor points on the binding site to avoid steric clashes due to the terminal methyl group of the ester function.

## 4. Conclusion

An *in-house* library of structurally diverse small molecules was screened with the aim of finding new c-Src inhibitors. It emerged that the most active compounds contained an indolinone core, which was selected as a promising scaffold for further investigations. Variously substituted 3-(hetero)arylideneindolin-2-ones were designed and synthesised to highlight the structural determinants for the activity. Most of the compounds showed a percentage of inhibition of the enzyme >70% at 100 μM and some of them also had a significant activity at 10 μM. Selected molecules were assayed on the human MCF-7 breast cancer cell line, showing moderate antiproliferative activity. Among the tested compounds, **49** demonstrated cytotoxicity comparable to that of the reference compound Dasatinib, but lacked any significant activity in the cell-based assay, indicating the possibility that some off-target effect could take place. Conversely, **34**, which showed the best inhibition profile towards the isolated enzyme, resulted ineffective (IC_50_>100 μM) on the cell line. This finding likely depends on pharmacokinetic issues probably related to the difficulty of the compound to reach the intracellular target. Therefore, further studies will be necessary to improve the pharmacological profile of this class of compounds. The results of molecular docking studies, which were performed to propose a binding mode within the c-Src binding site, will guide and complement future activities.

## Supplementary Material

Supplemental Material
