# Correction

**DOI:** 10.1080/14756366.2022.2133503

**Published:** 2022-10-13

**Article title:** Synthesis and biological activity evaluation of 3-(hetero) arylideneindolin-2-ones as potential c-Src inhibitors

**Authors:** Salvatore Princiotto, Loana Musso, Fabrizio Manetti, Valentina Marcellini, Giovanni Maga, Emmanuele Crespan, Cecilia Perini, Nadia Zaffaroni, Giovanni Luca Beretta, and Sabrina Dallavalle

**Journal:** *Journal of Enzyme Inhibition and Medicinal Chemistry*

**Bibliometrics:** Volume 37, Number 1, pages 2382–2394

**DOI:** http://10.1080/14756366.2022.2117317

When the article has been published online, the wrong version of the Table has been indicated. Below is the correct version of the table.

**Table:** 

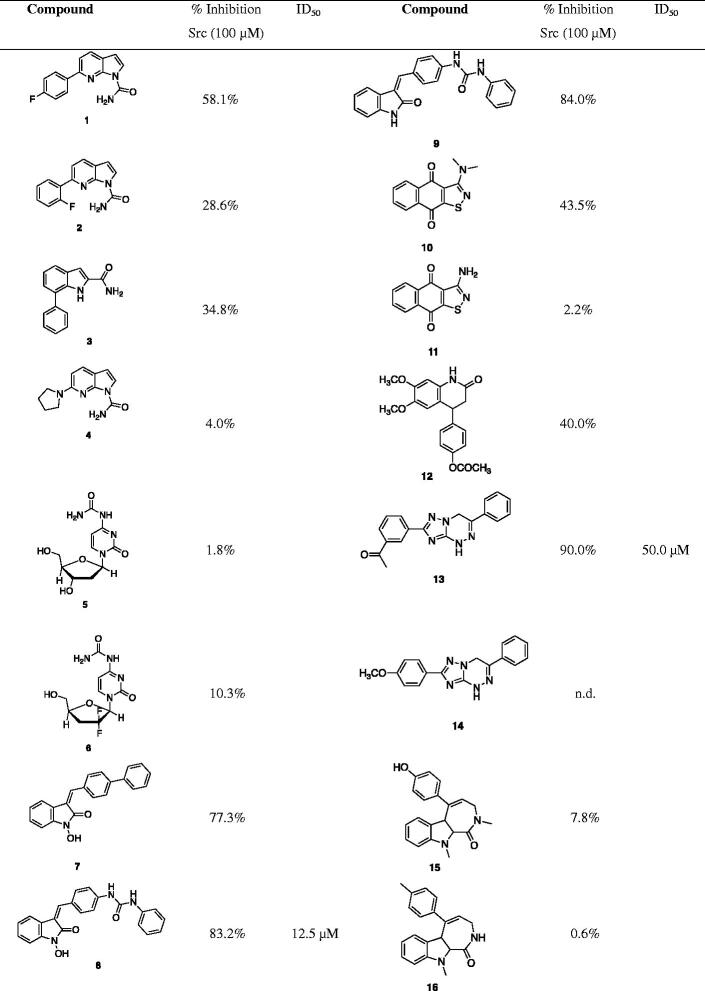

**Table:** 

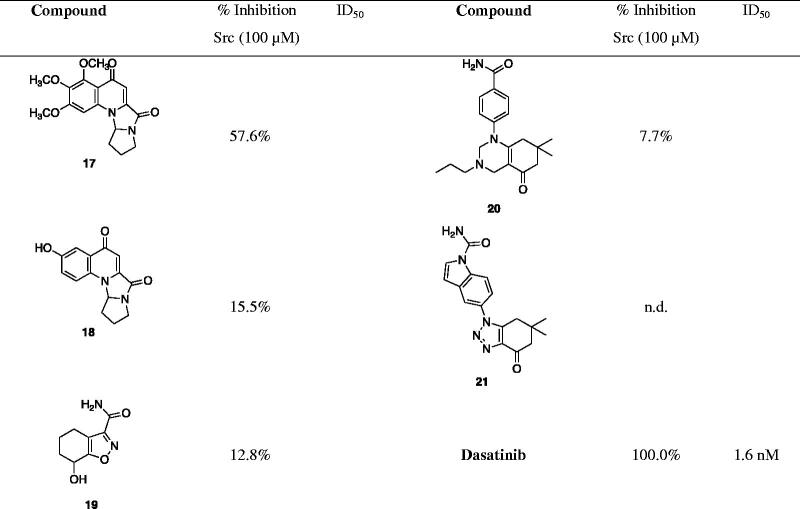

The online version has been corrected.

