# Supplementary material for: Synthesis and biological activity evaluation of 3-(hetero) arylideneindolin-2-ones as potential c-Src inhibitors
Source: J Enzyme Inhib Med Chem. 2022 Sep 1;37(1):2382–94. doi: 10.1080/14756366.2022.2117317 (PMC9448371; doi:10.1080/14756366.2022.2117317)
Supplement: Supplemental Material [file IENZ_A_2117317_SM8582.pdf]

Synthesis and biological activity evaluation of 3-(hetero) arylideneindolin-2-ones as potential c-Src inhibitors

Salvatore Princiotto,[a*] Loana Musso,[a] Fabrizio Manetti,[b] Valentina Marcellini,[b] Giovanni Maga,[c] Emmanuele Crespan,[c] Cecilia Perini,[c] Nadia Zaffaroni,[d] Giovanni Luca Beretta,[d] and Sabrina Dallavalle.[a]

[a] Department of Food, Environmental and Nutritional Sciences (DeFENS), University of Milan, Via Celoria 2, 20133 Milan, Italy; [b] Dipartimento di Biotecnologie, Chimica e Farmacia, Dipartimento di Eccellenza 2018-2022, Università di Siena, I-53100 Siena, Italy; [c] Institute of Molecular Genetics IGM-CNR "Luigi Luca Cavalli-Sforza", via Abbiategrasso 207, I-27100 Pavia, Italy; [d] Molecular Pharmacology Unit, Department of Applied Research and Technological Development, Fondazione IRCCS Istituto Nazionale Tumori, Via Amadeo 42, 20133 Milan, Italy.

*corresponding author: salvatore.princiotto@unimi.it

Supplementary Material

Table of contents

Figure S1 **S2**

Figure S2 **S2**

^{1}H NMR and ^{13}C NMR spectra of synthesised compounds **S3-S21**

Information Classification: General

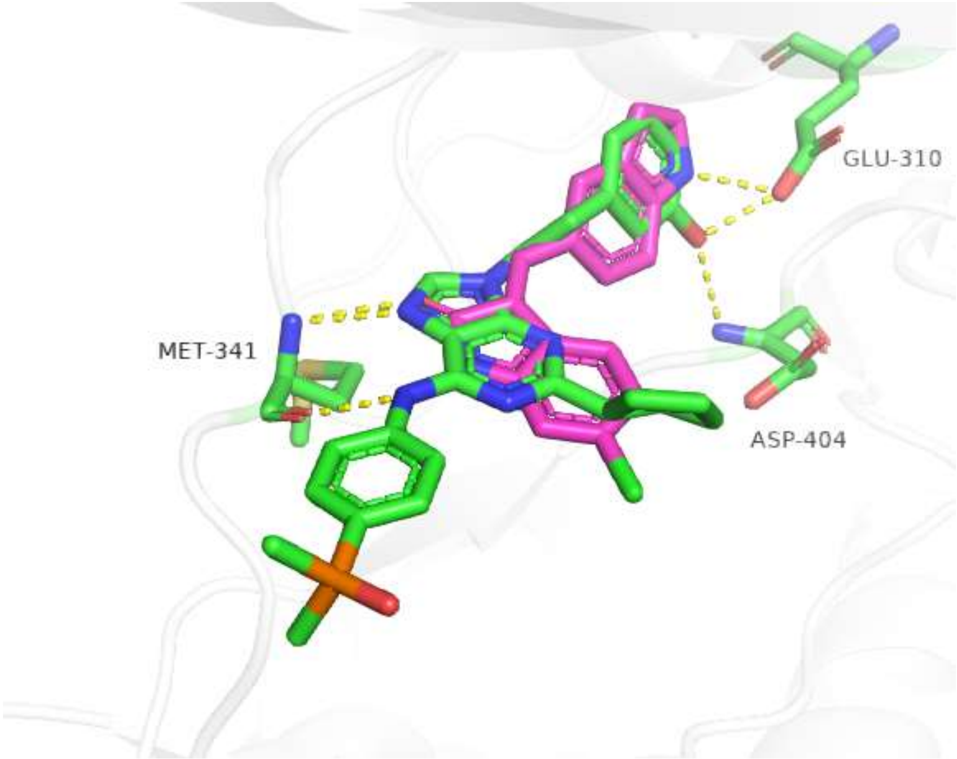

Figure S1. Graphical representation of the binding pose of **50** (magenta) within the c-Src ATP binding site, in comparison to the co-crystallized inhibitor **AP23464** (green). Yellow dashed lines represent hydrogen bonds between Met341, Glu310, Asp404, and both ligands.

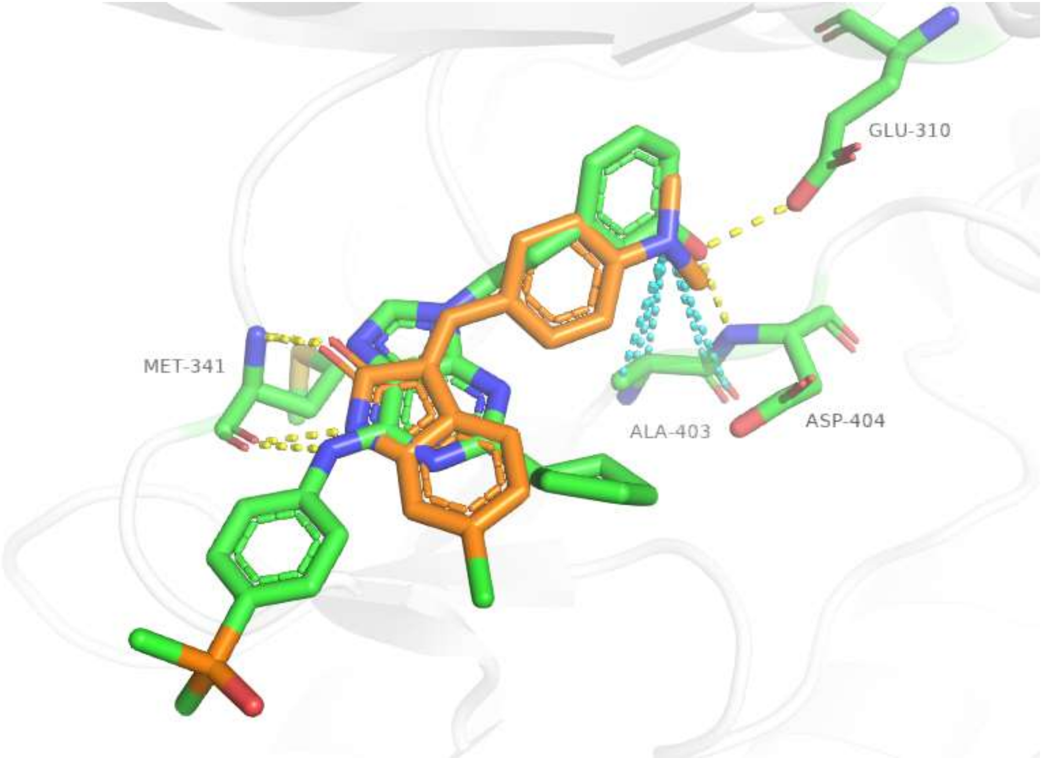

Figure S2. Graphical representation of the binding pose of **34** (brown) within the c-Src ATP binding site, in comparison to the co-crystallized inhibitor **AP23464** (green). Yellow dashed lines represent hydrogen bonds between Met341, Glu310, Ala403, Asp404, and both ligands. Cyan dashed lines show additional contacts (hydrophobic, in particular) between the ligand and the target.

 Information Classification: General

^{1}H NMR (600 MHz, DMSO-d_6) of **22**

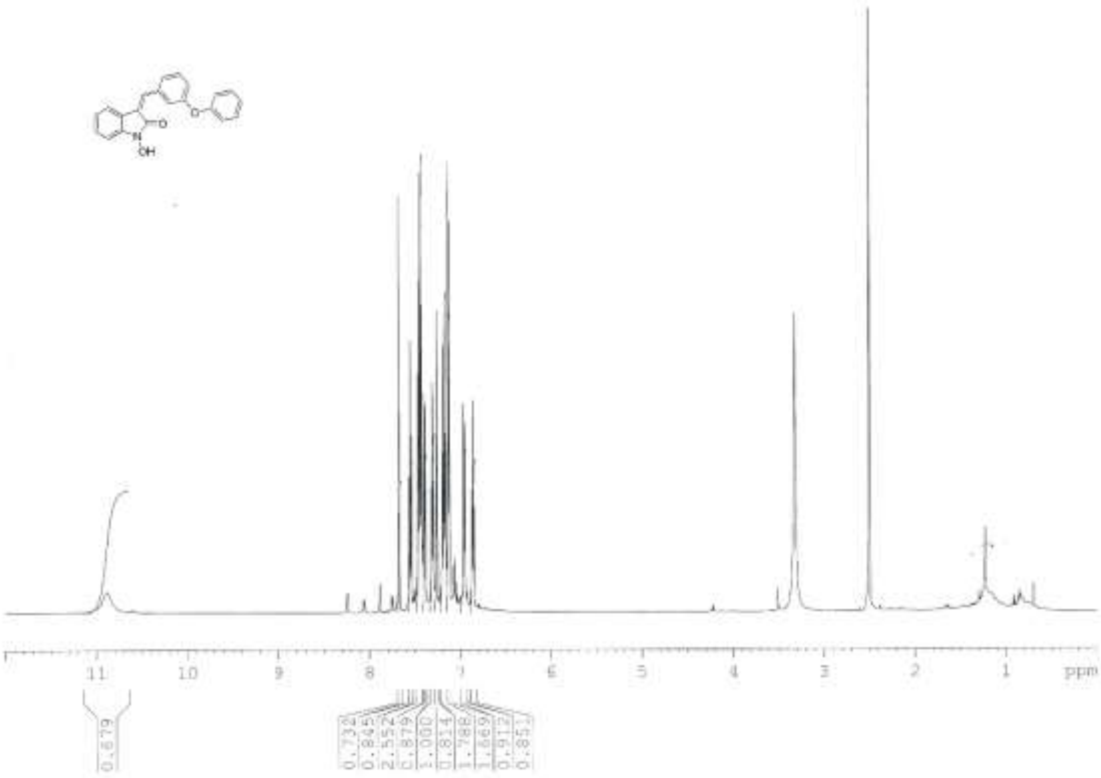

^{13}C NMR (150 MHz, DMSO-d_6) of **22**

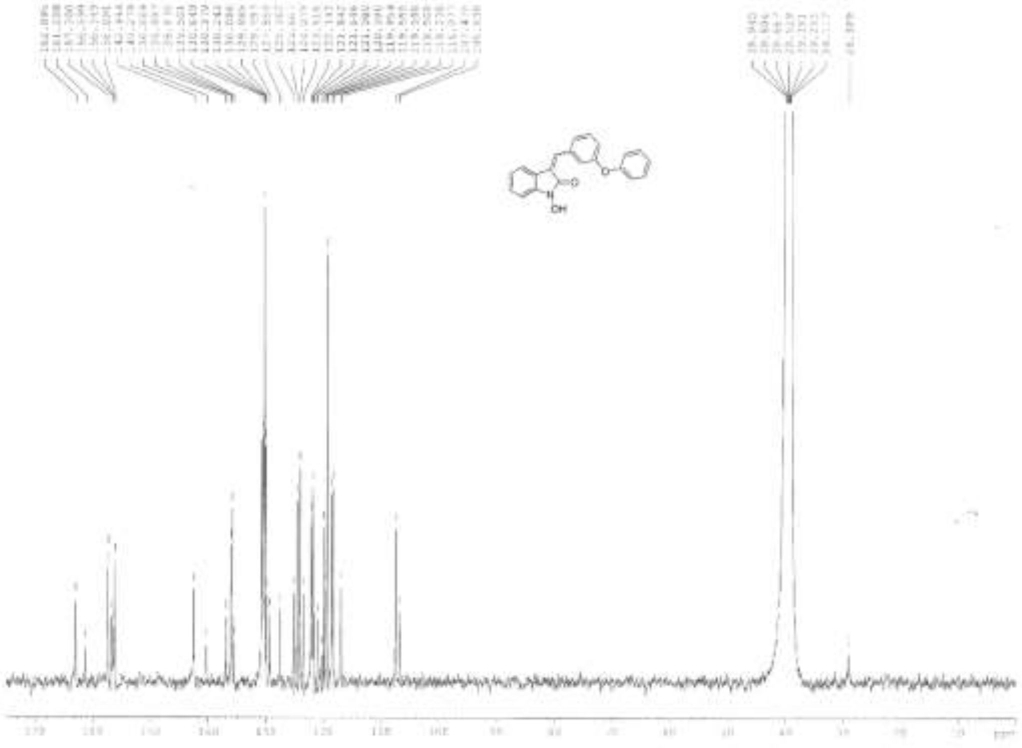

 Information Classification: General

^{1}H NMR (600 MHz, DMSO-d_6) of **27**

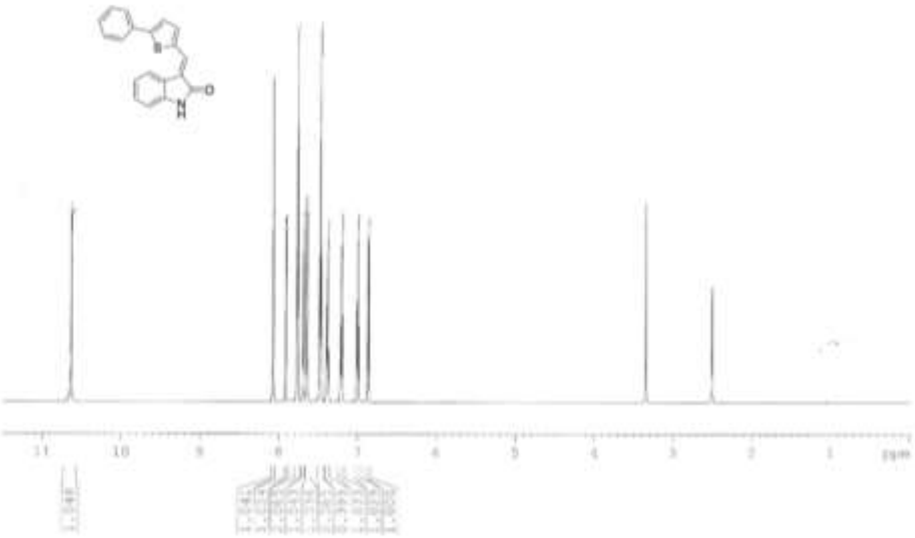

^{13}C NMR (150 MHz, DMSO-d_6) of **27**

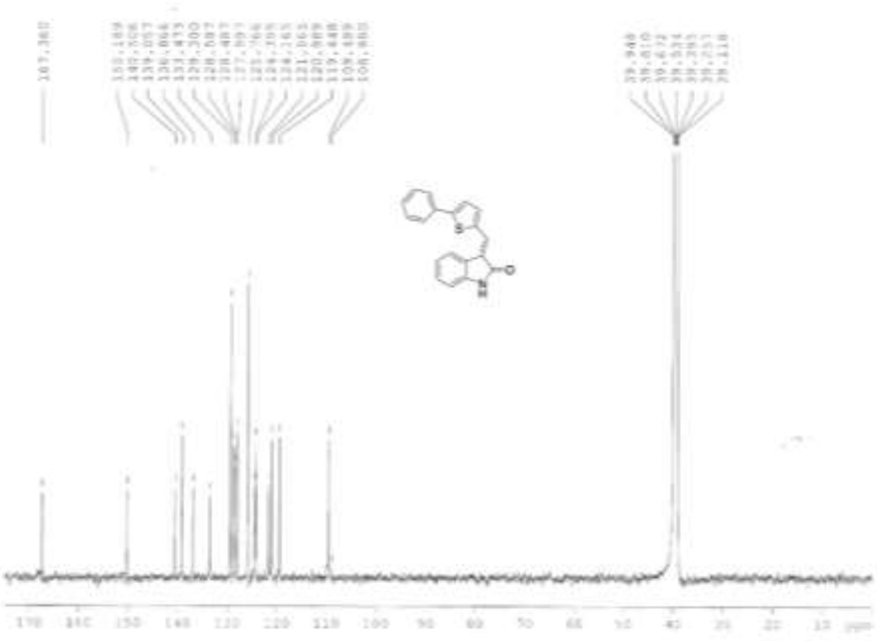

^{1}H NMR (600 MHz, DMSO-d_6) of **29**

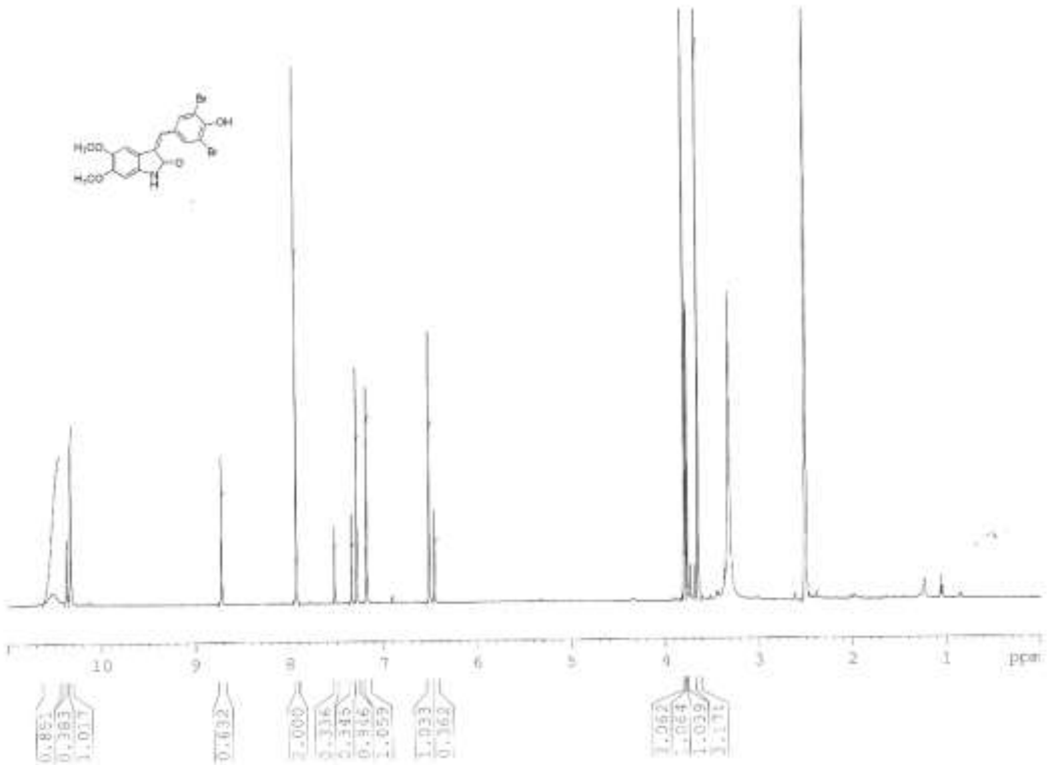

^{13}C NMR (150 MHz, DMSO-d_6) of **29**

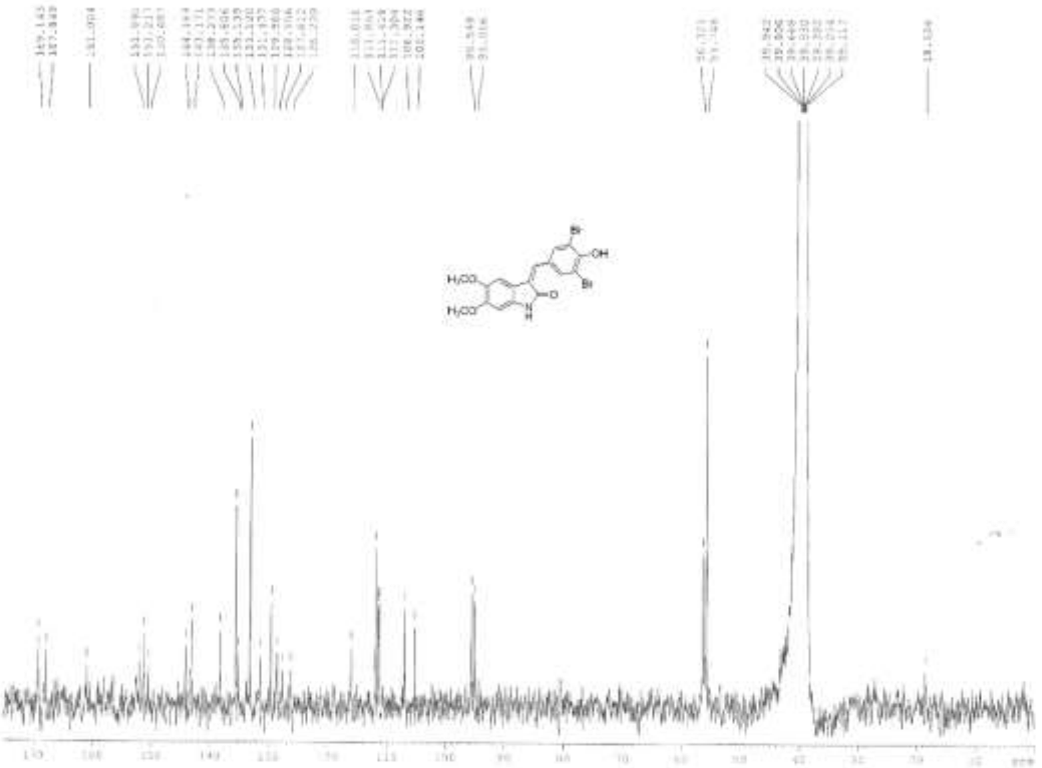

 Information Classification: General

^{1}H NMR (600 MHz, DMSO-d_6) of **30**

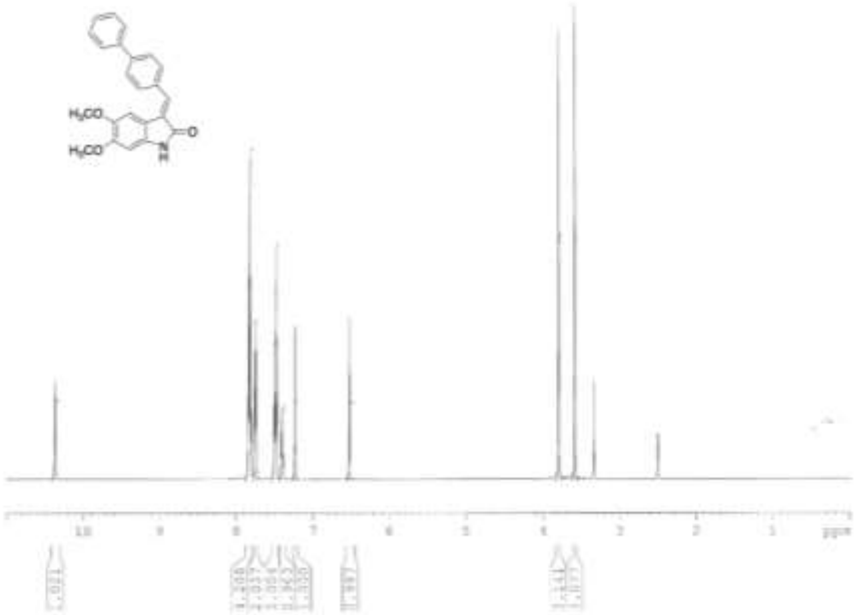

^{13}C NMR (150 MHz, DMSO-d_6) of **30**

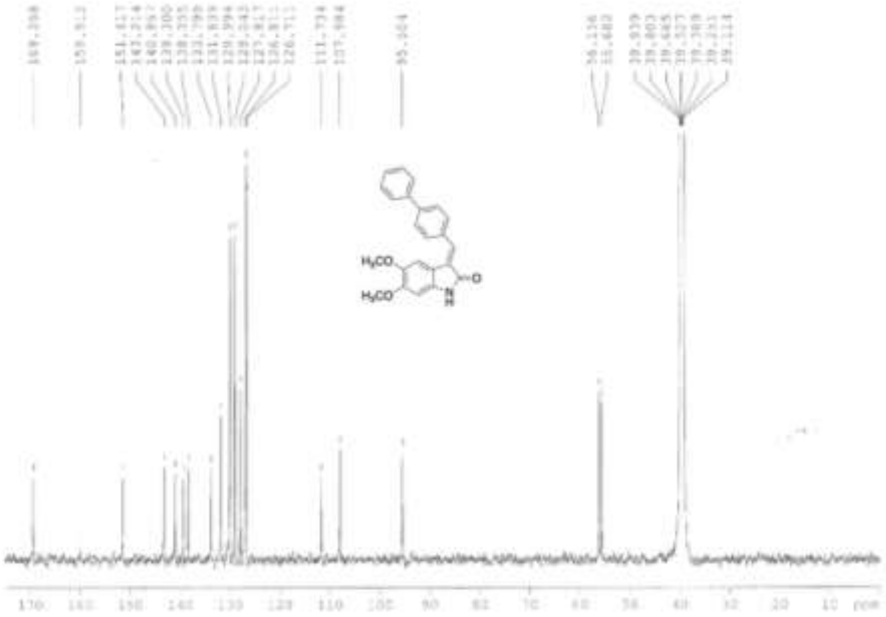

^{1}H NMR (600 MHz, DMSO-d_6) of **32**

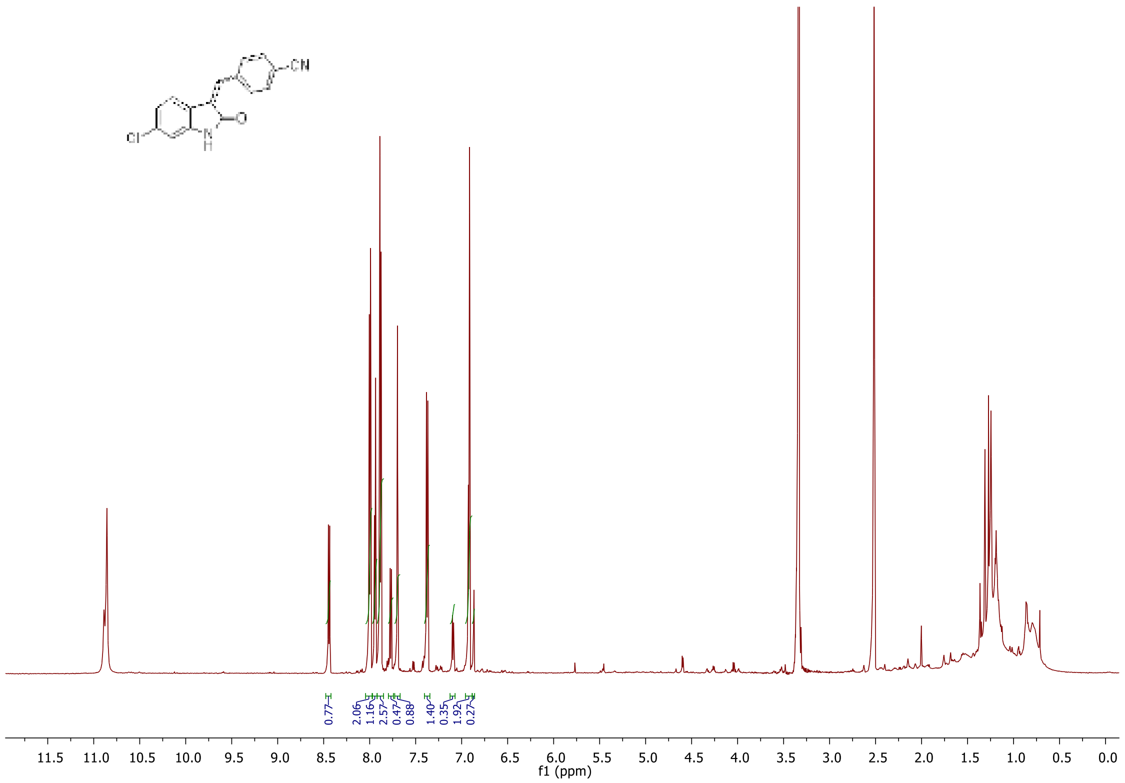

^{13}C NMR (150 MHz, DMSO-d_6) of **32**

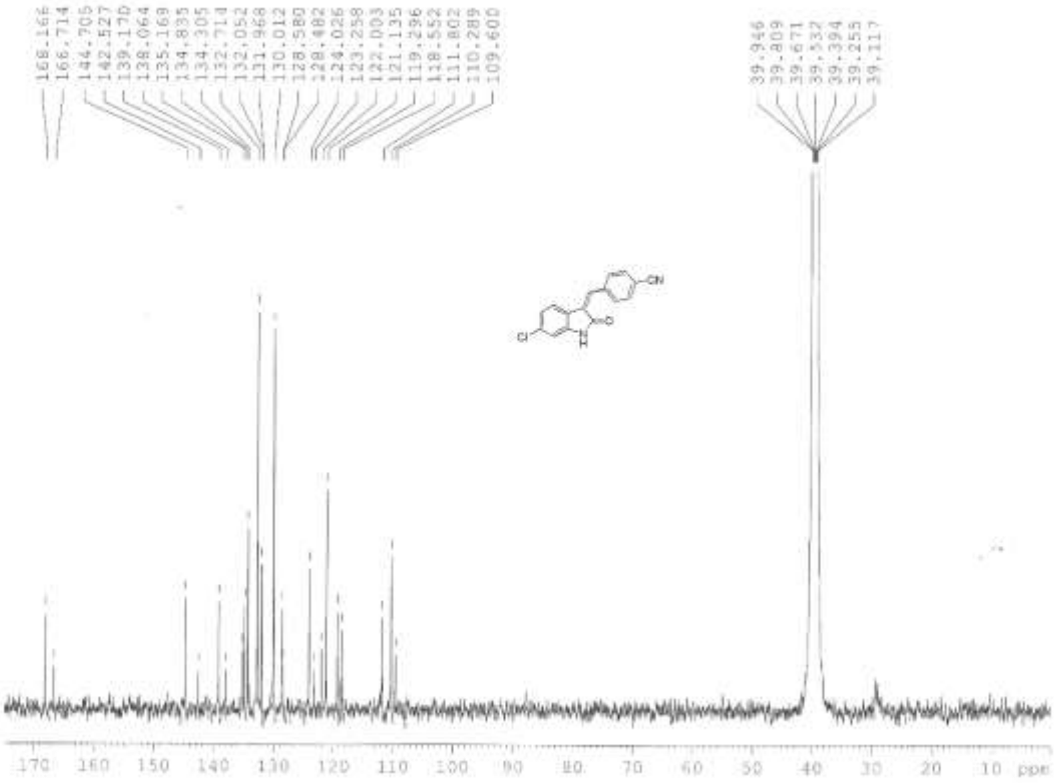

 Information Classification: General

^{1}H NMR (600 MHz, DMSO-d_6) of **33**

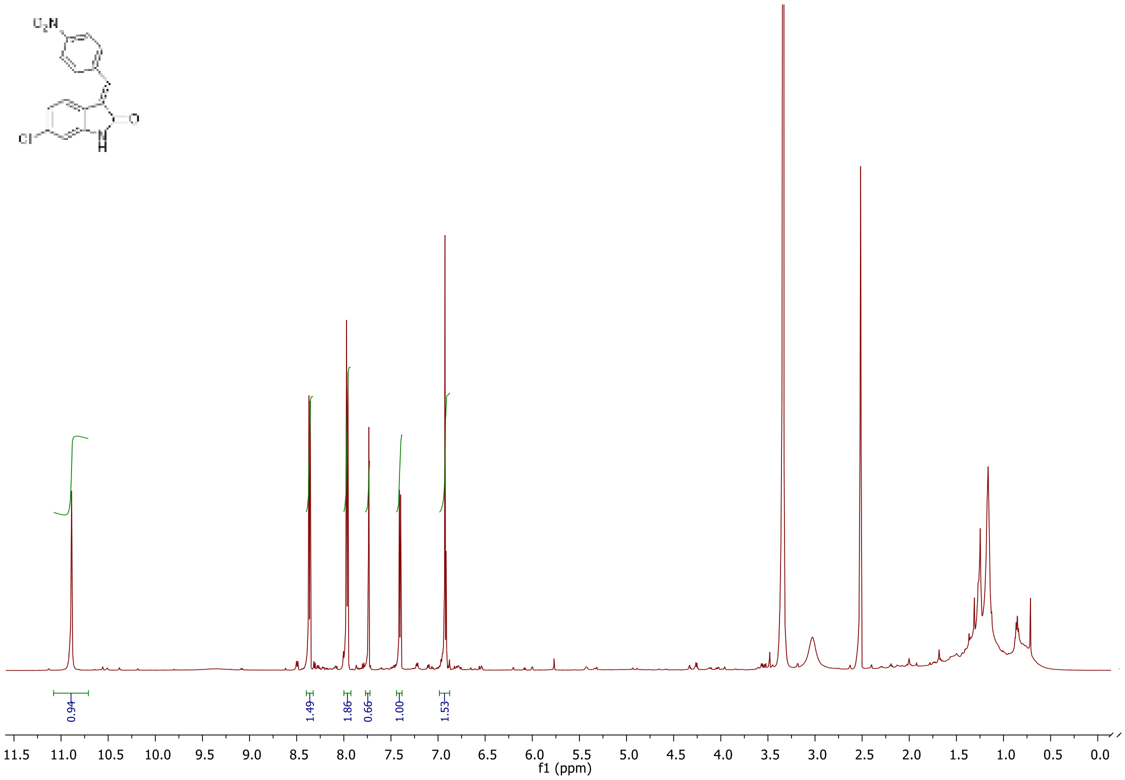

^{13}C NMR (150 MHz, DMSO-d_6) of **33**

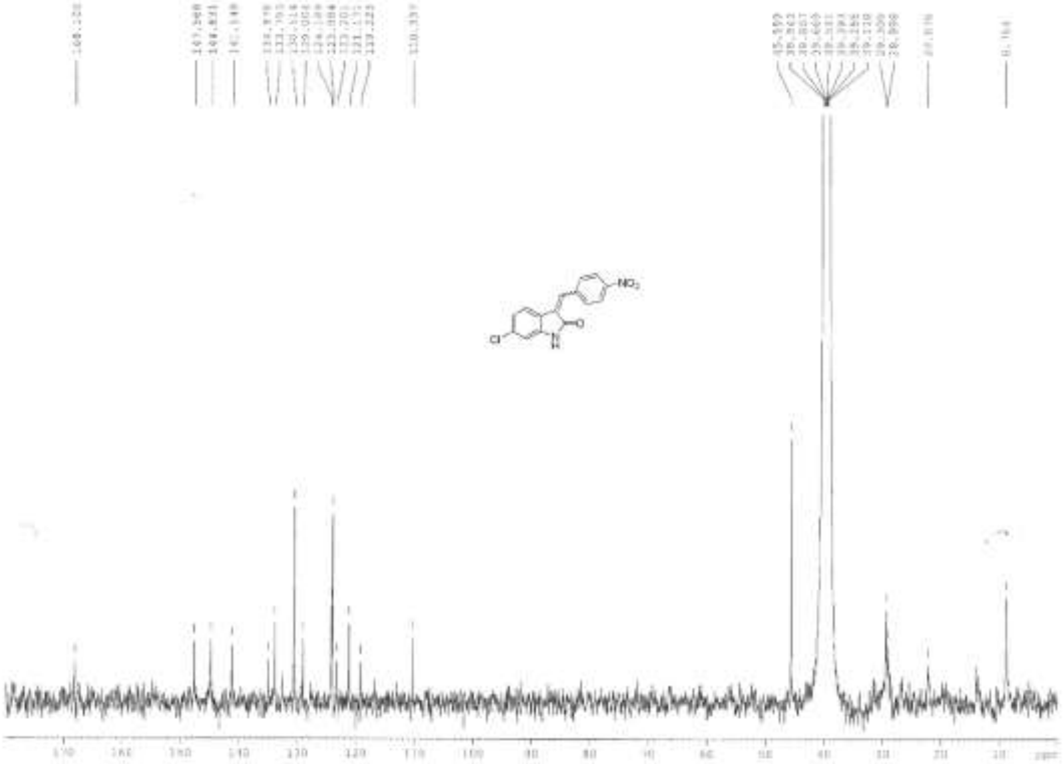

^{1}H NMR (600 MHz, DMSO-d_6) of **34**

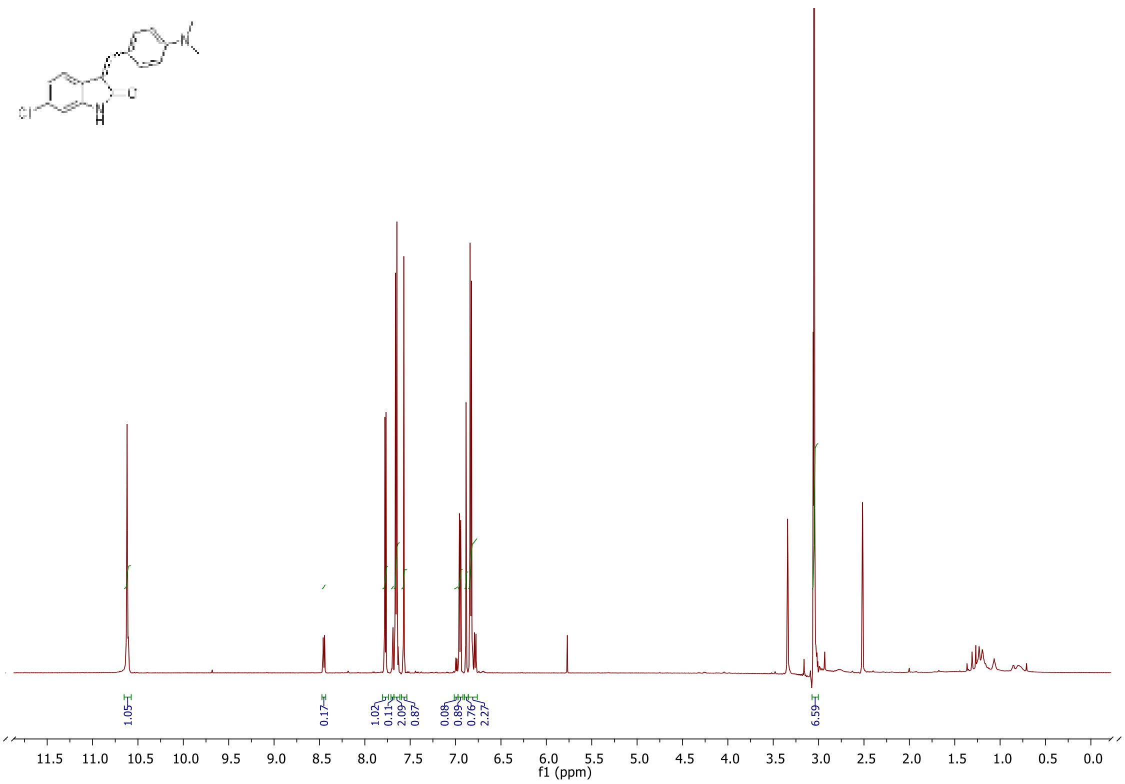

^{13}C NMR (150 MHz, DMSO-d_6) of **34**

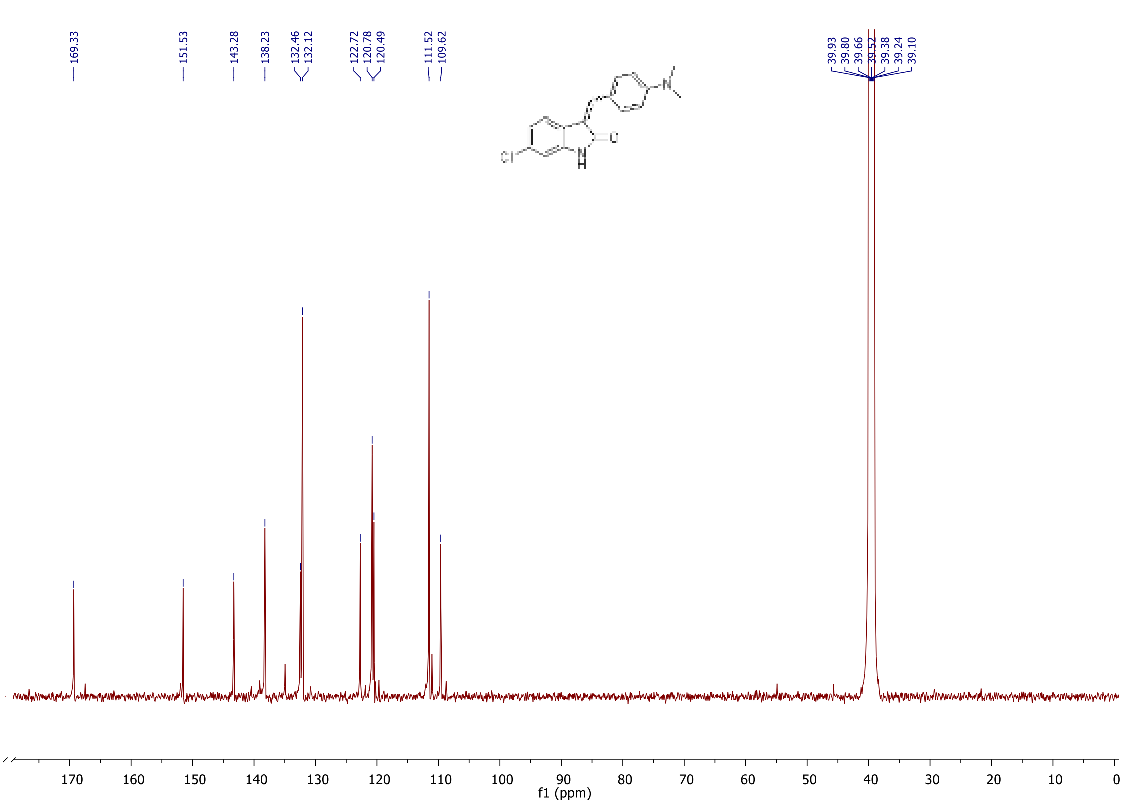

 Information Classification: General

^{1}H NMR (600 MHz, DMSO-d_6) of **35**

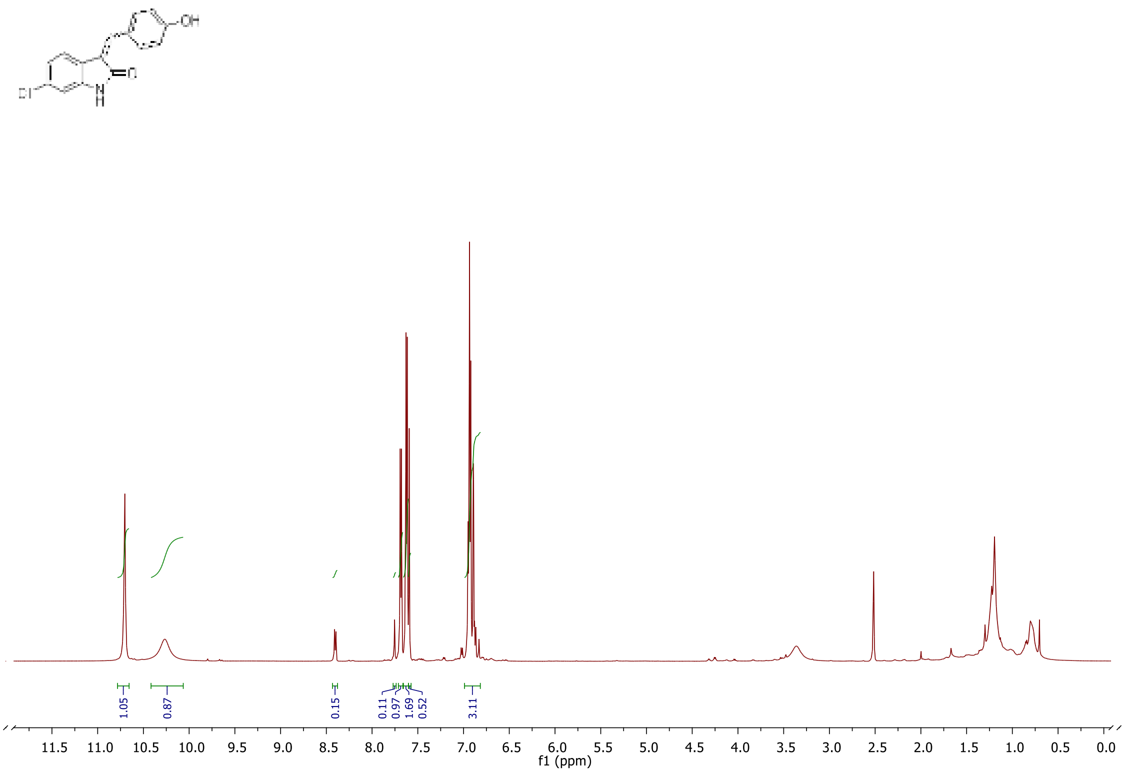

^{13}C NMR (150 MHz, DMSO-d_6) of **35**

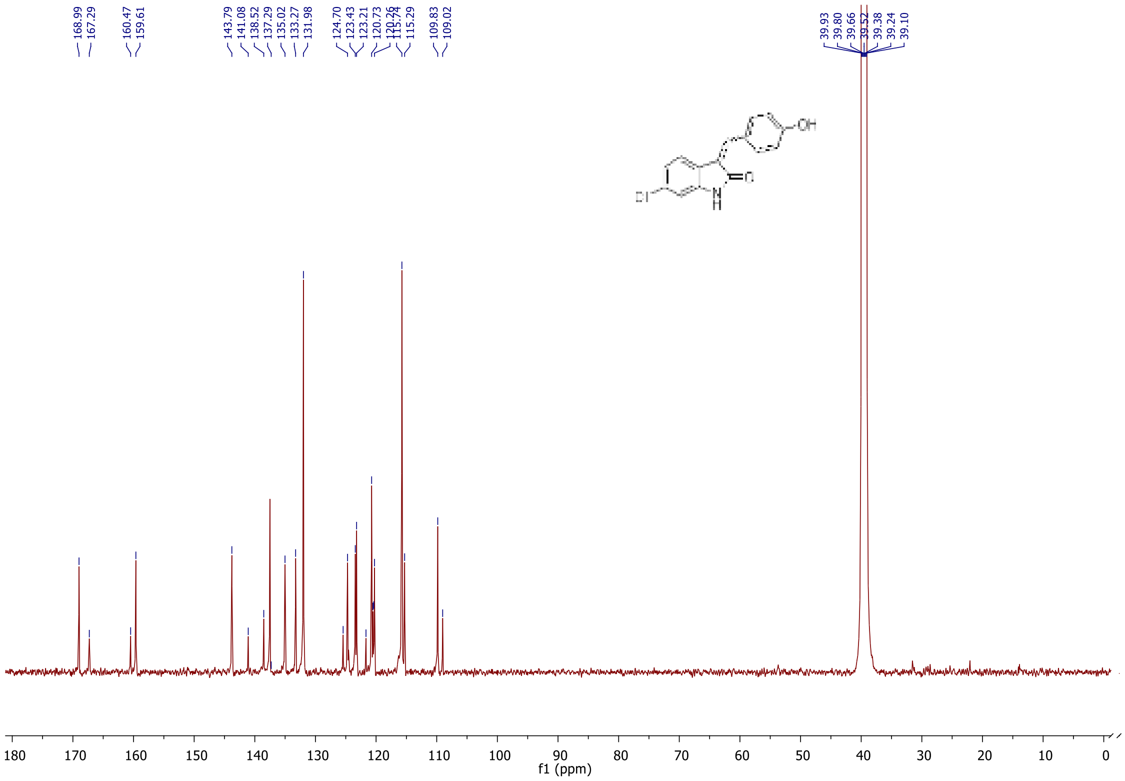

Information Classification: General

^{1}H NMR (600 MHz, DMSO-d_6) of **36**

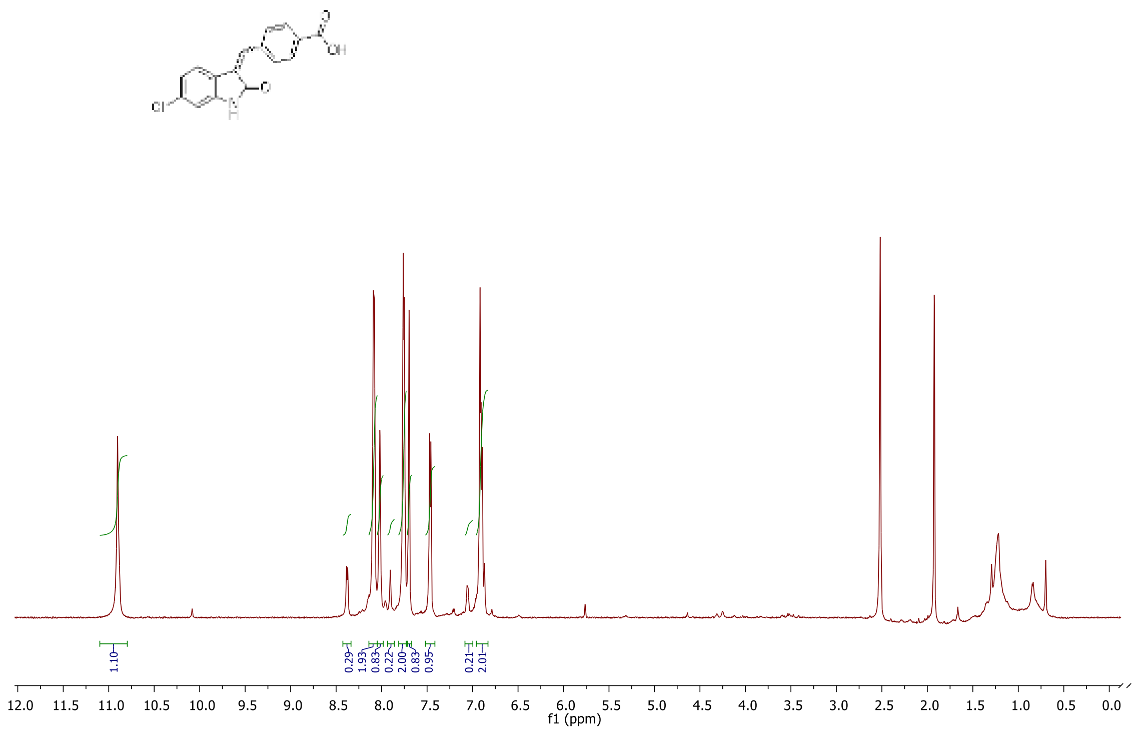

^{13}C NMR (150 MHz, DMSO-d_6) of **36**

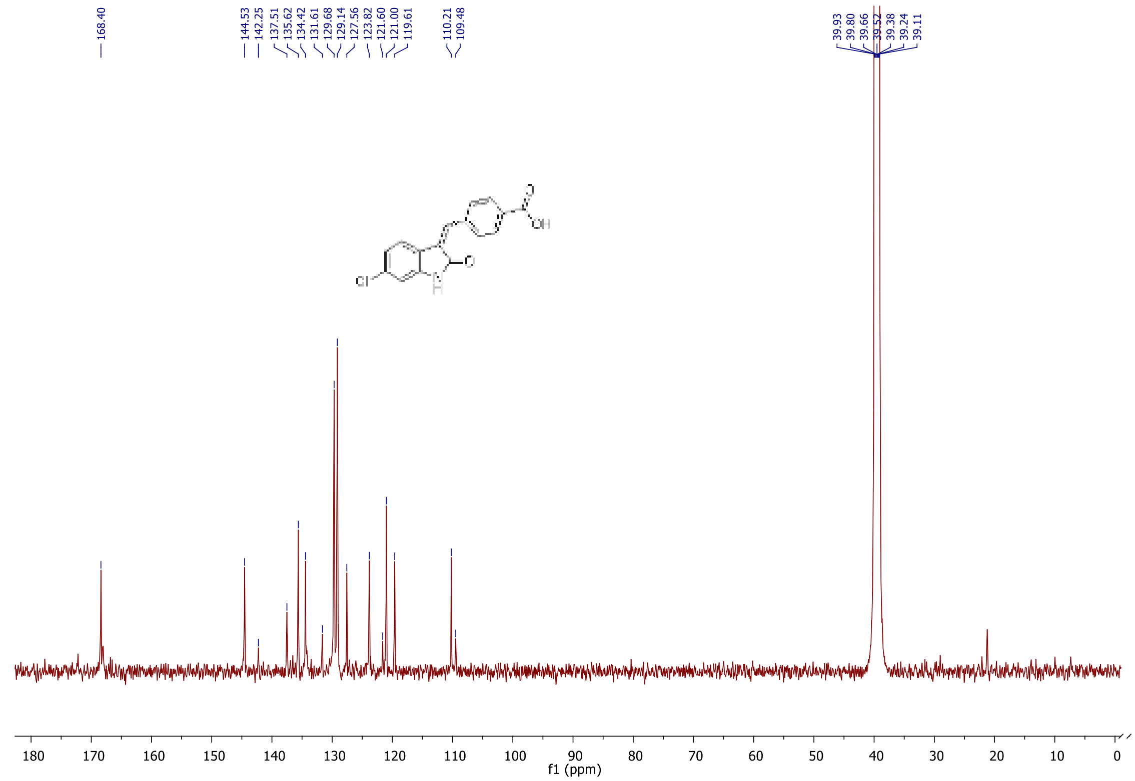

Information Classification: General

^{1}H NMR (600 MHz, DMSO-d_6) of **37**

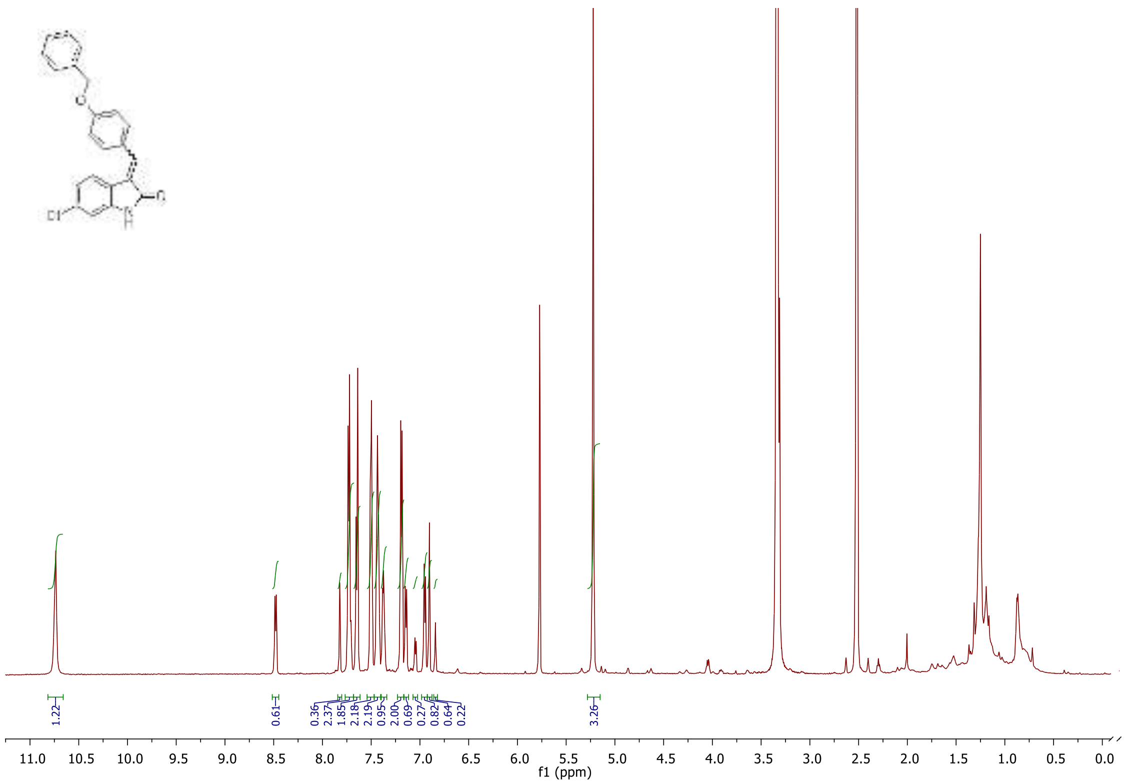

^{13}C NMR (150 MHz, DMSO-d_6) of **37**

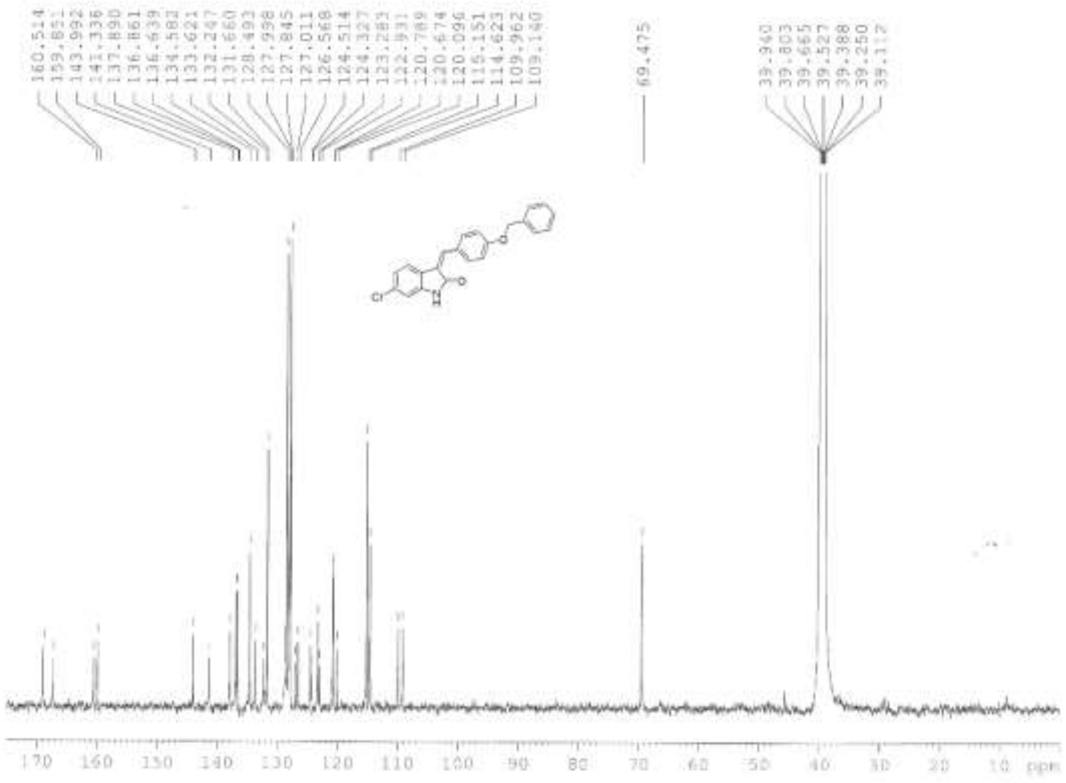

Information Classification: General

^{1}H NMR (600 MHz, DMSO-d_6) of **38**

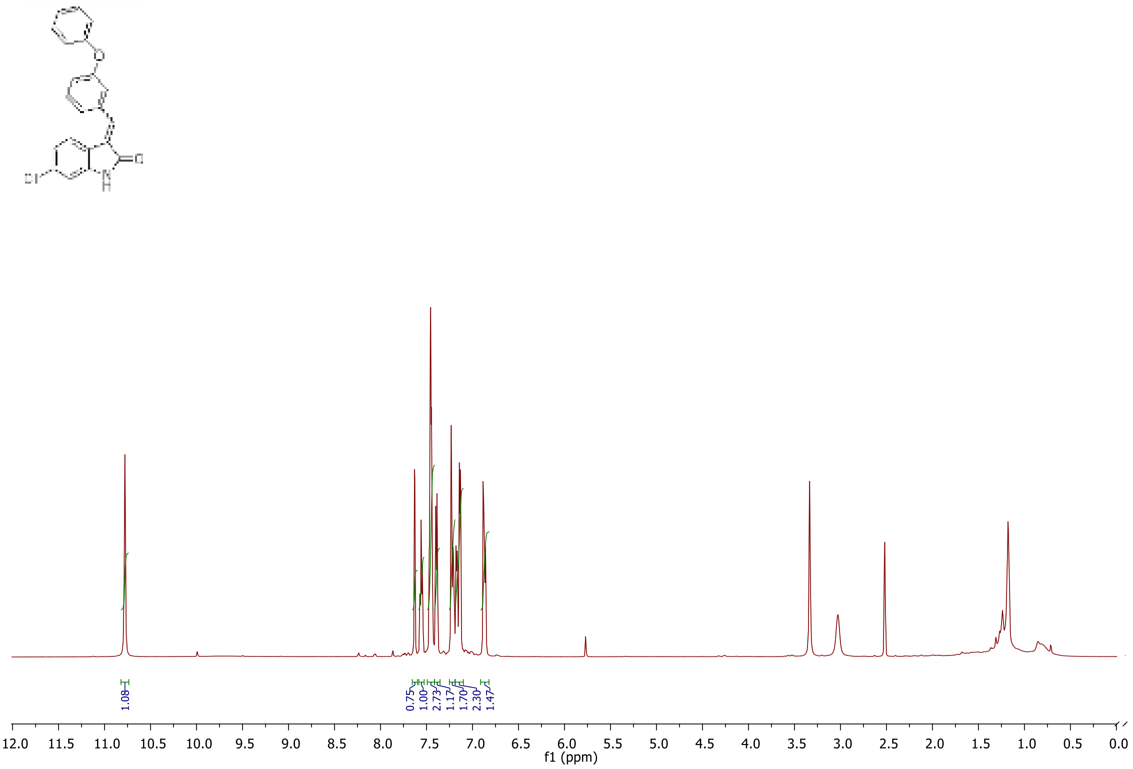

^{13}C NMR (150 MHz, DMSO-d_6) of **38**

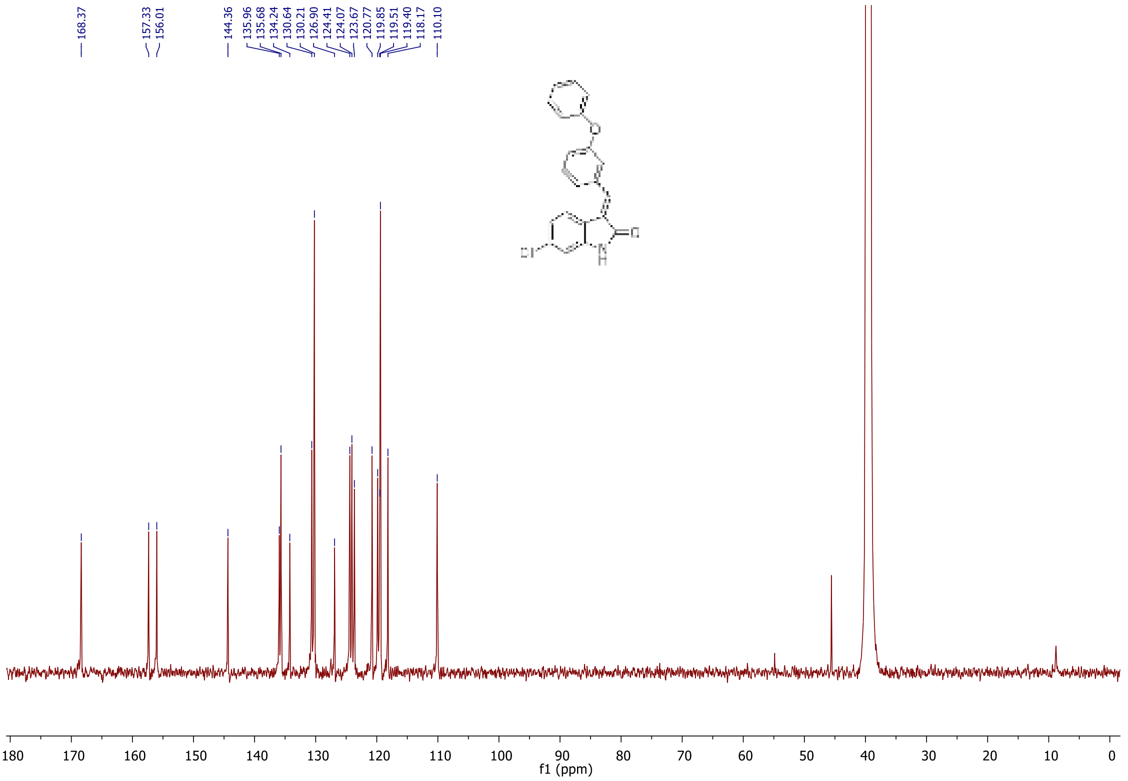

Information Classification: General

^{1}H NMR (600 MHz, DMSO-d_6) of **43**

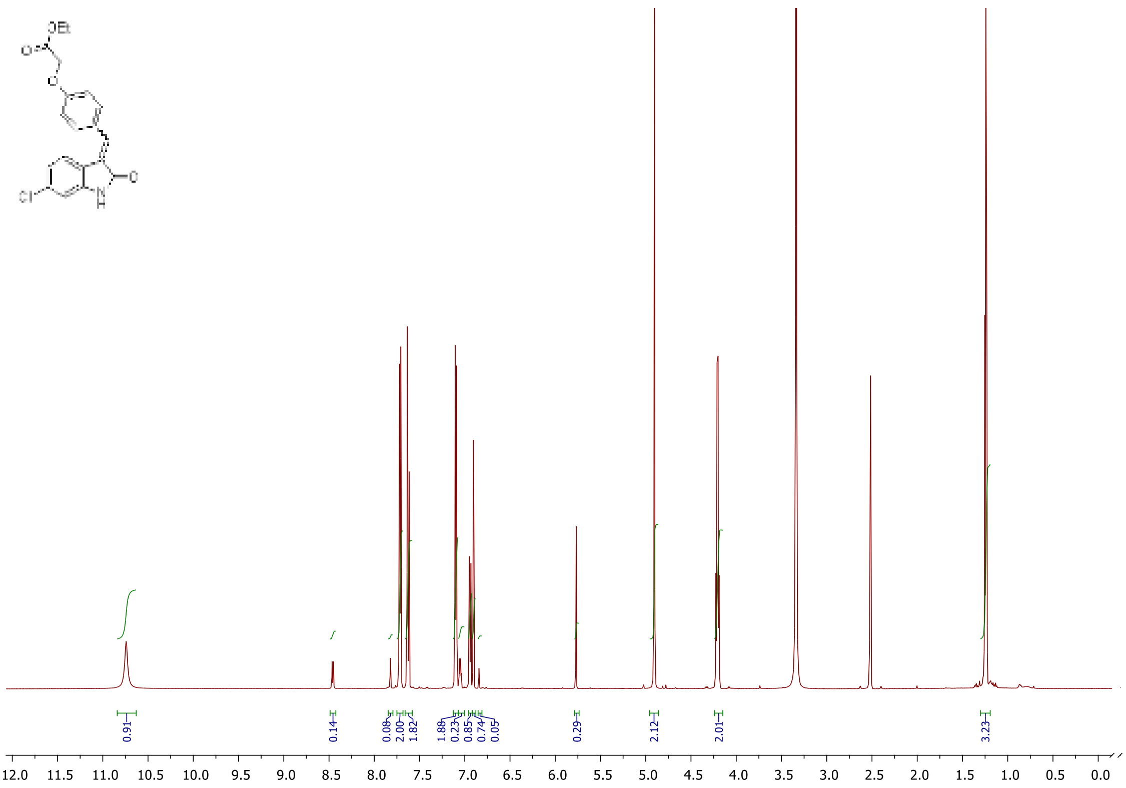

^{13}C NMR (150 MHz, DMSO-d_6) of **43**

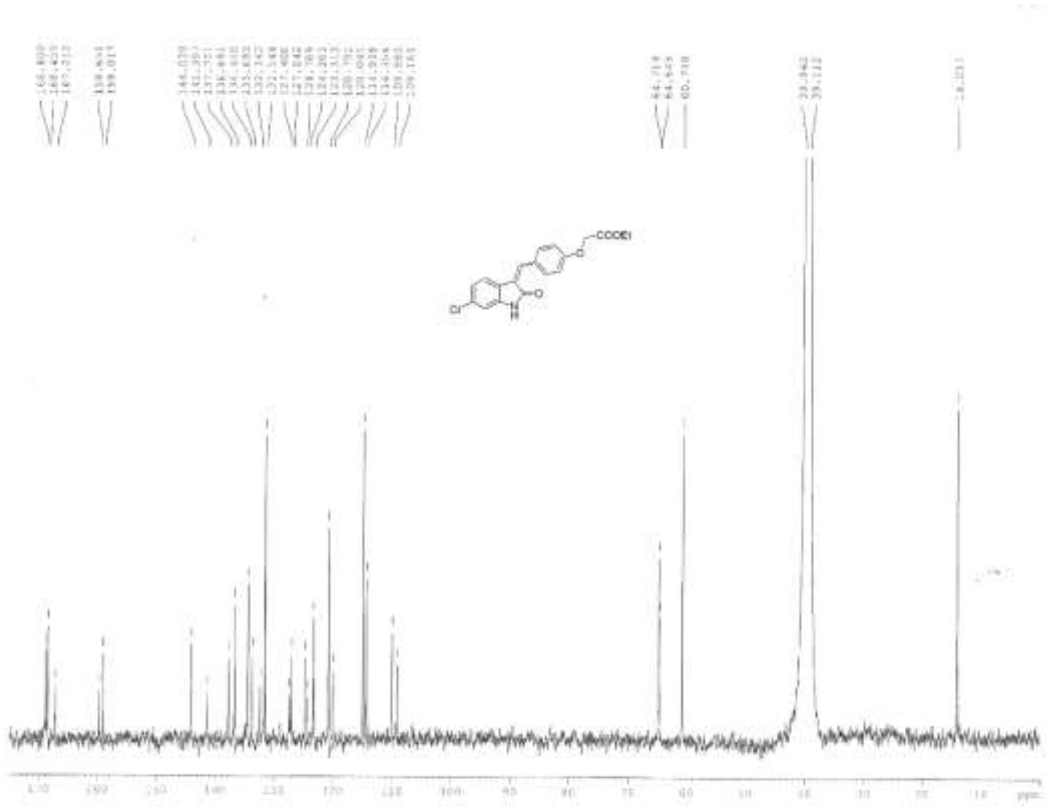

Information Classification: General

^{1}H NMR (600MHz, DMSO-d_6) of **44**

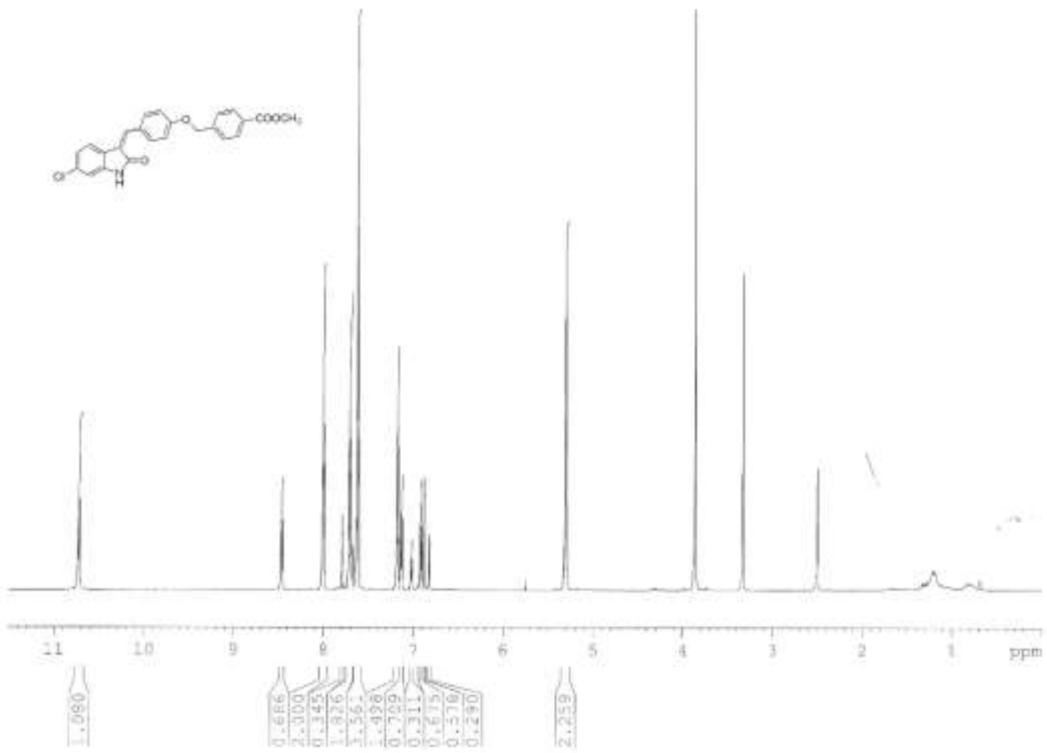

^{13}C NMR (150 MHz, DMSO-d_6) of **44**

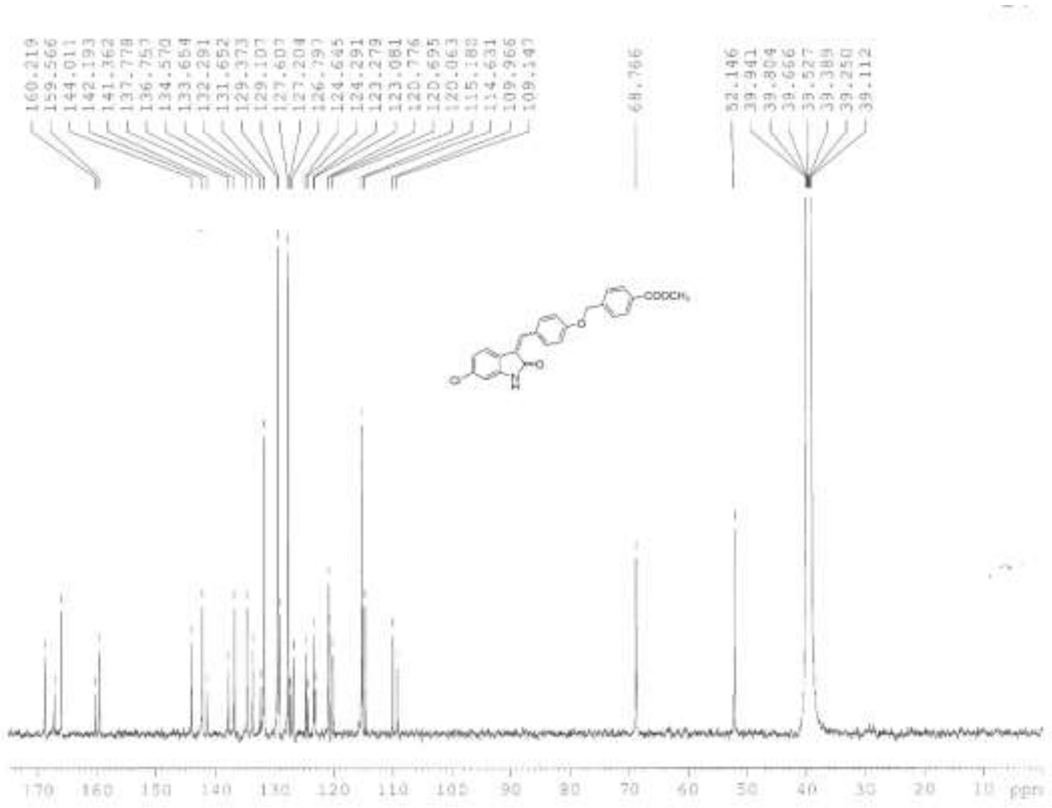

Information Classification: General

^{1}H NMR (600 MHz, DMSO-d_6) of **45**

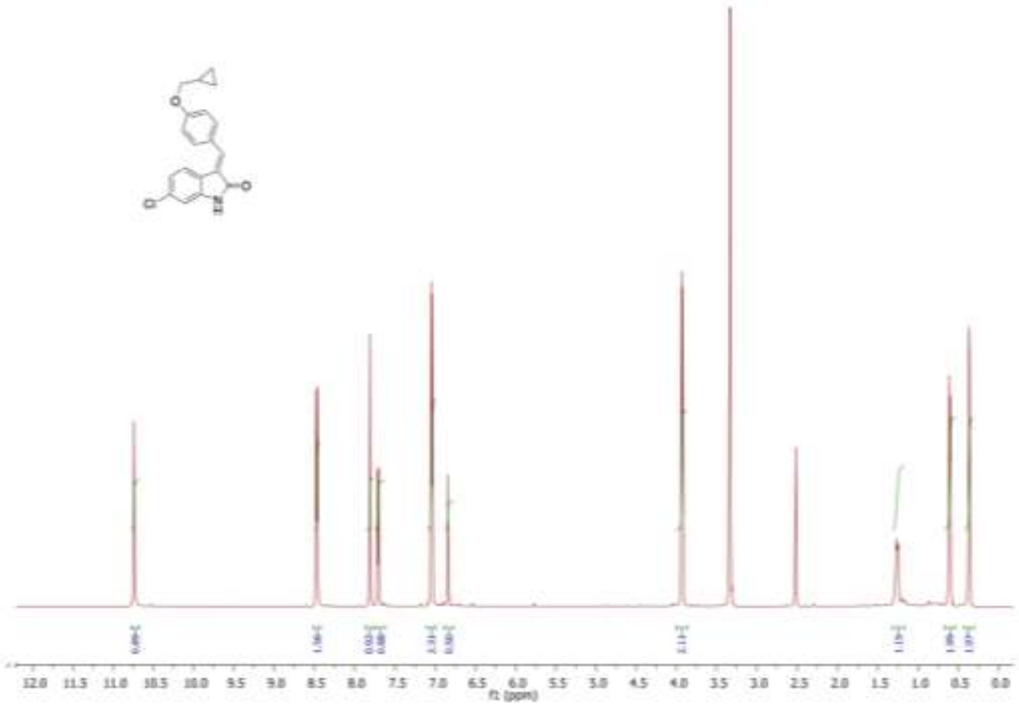

^{13}C NMR (150 MHz, DMSO-d_6) of **45**

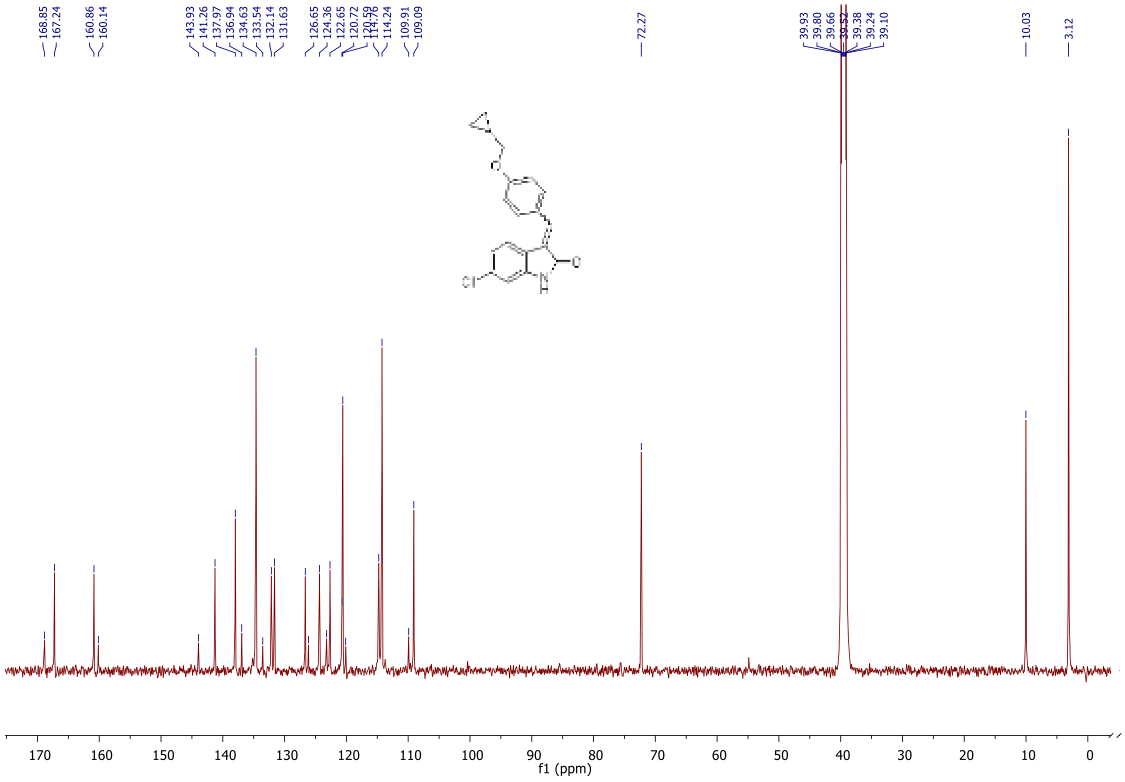

Information Classification: General

^{1}H NMR (600 MHz, DMSO-d_6) of **46**

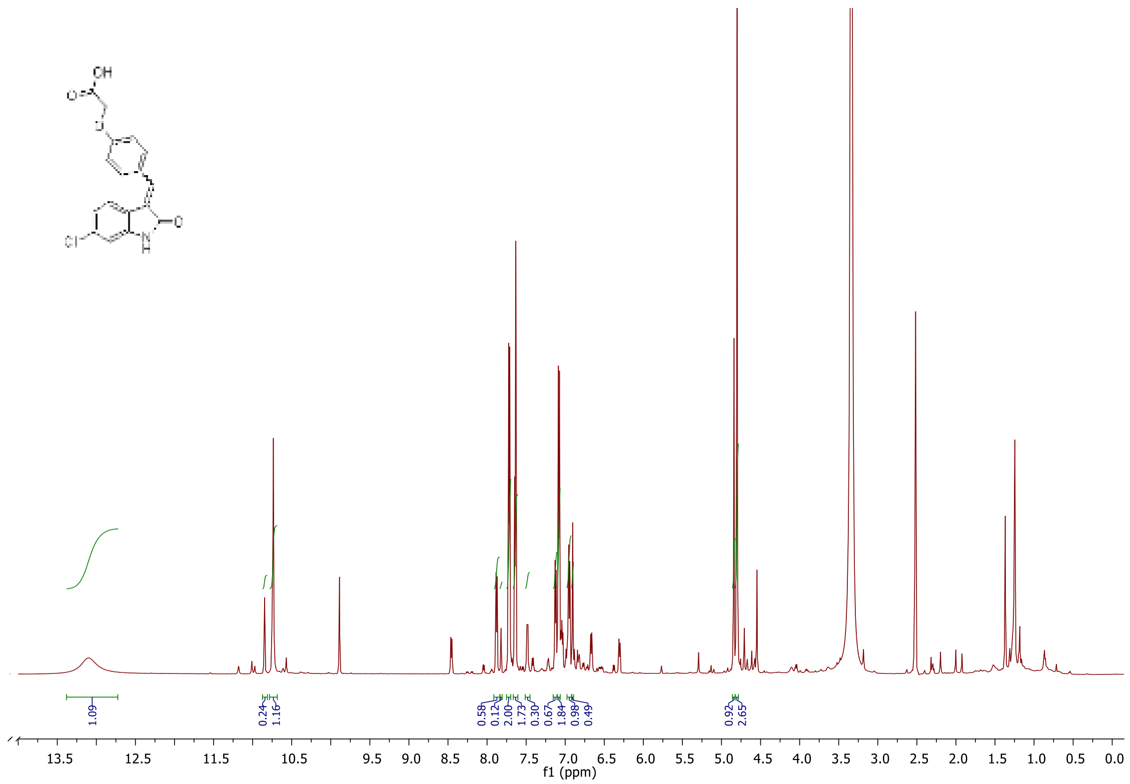

^{13}C NMR (150 MHz, DMSO-d_6) of **46**

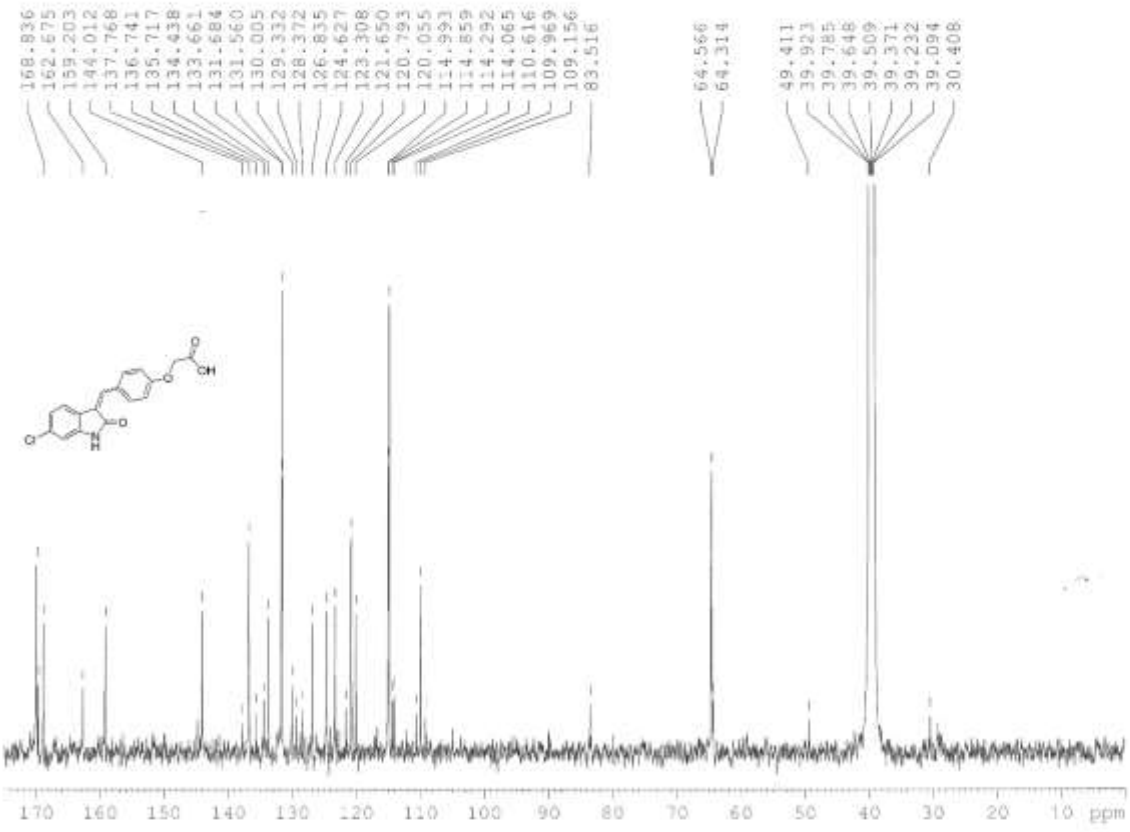

Information Classification: General

^{1}H NMR (600 MHz, DMSO-d_6) of **47**

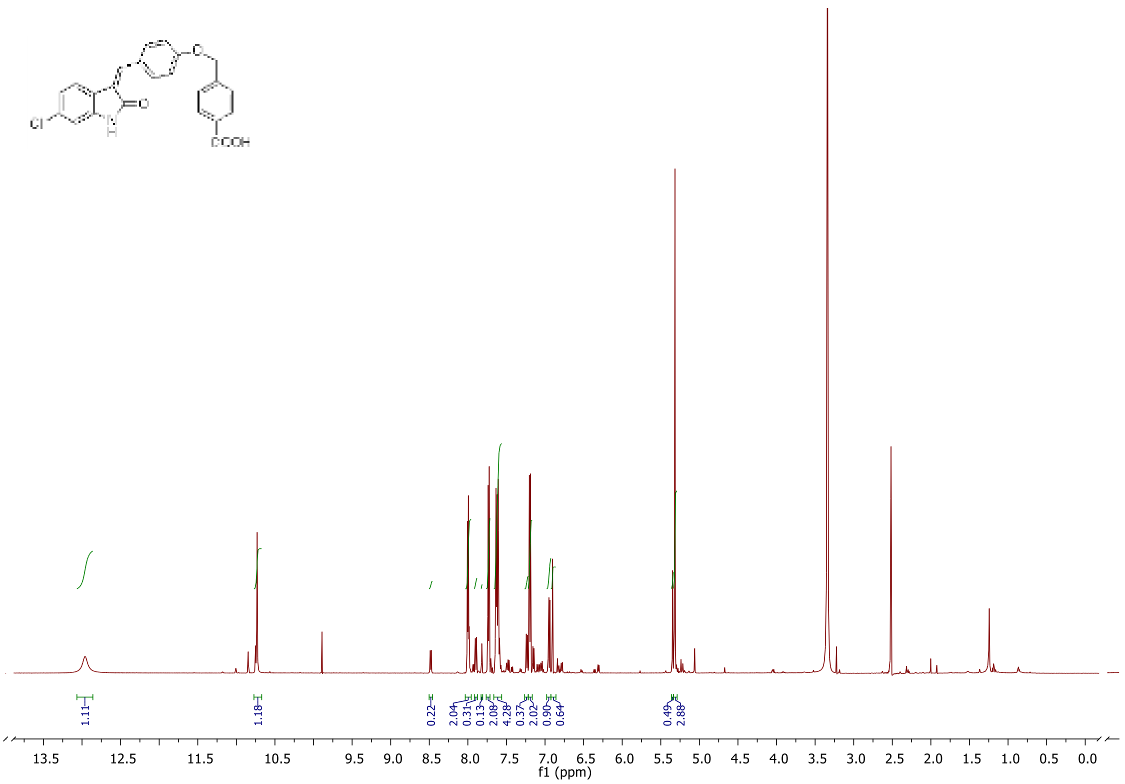

^{13}C NMR (150 MHz, DMSO-d_6) of **47**

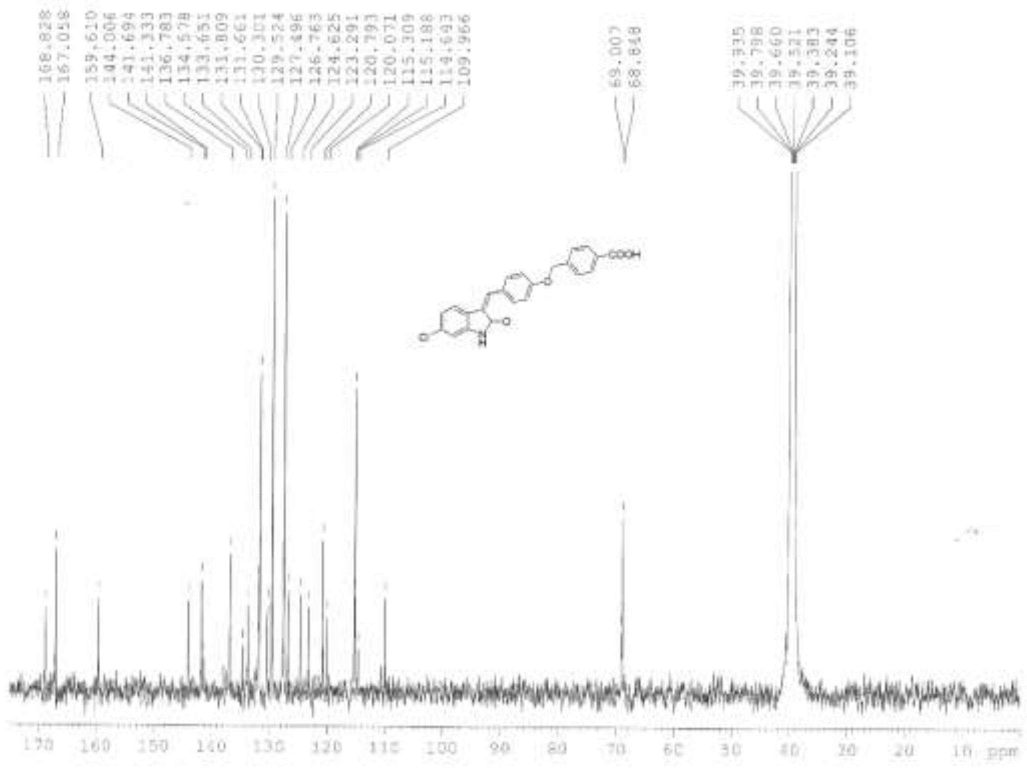

Information Classification: General

^{1}H NMR (600 MHz, DMSO-d_6) of **48**

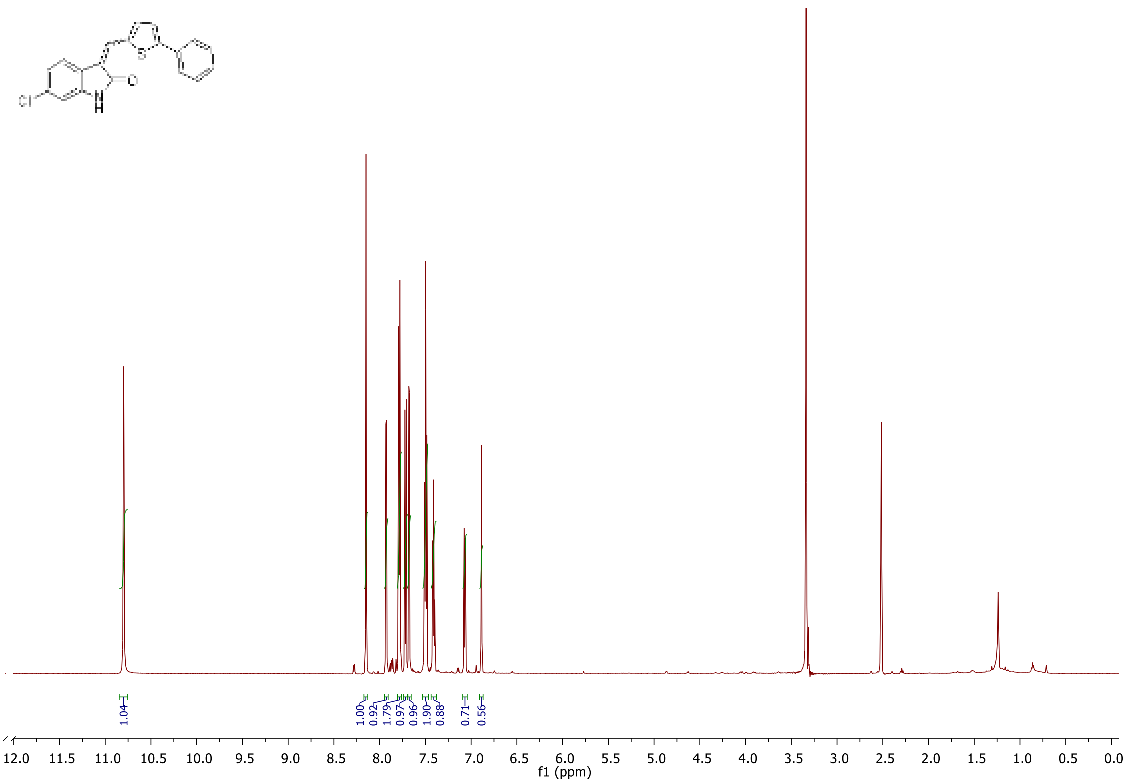

^{13}C NMR (150 MHz, DMSO-d_6) of **48**

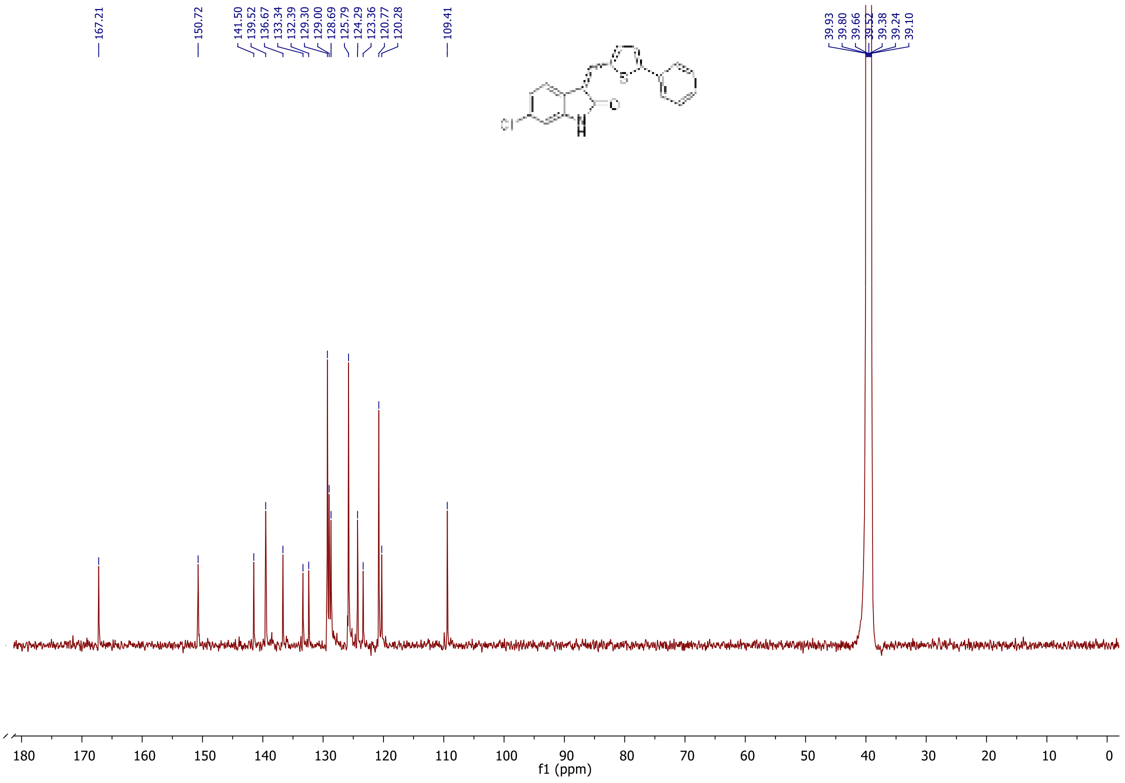

Information Classification: General

^{1}H NMR (600 MHz, DMSO-d_6) of **49**

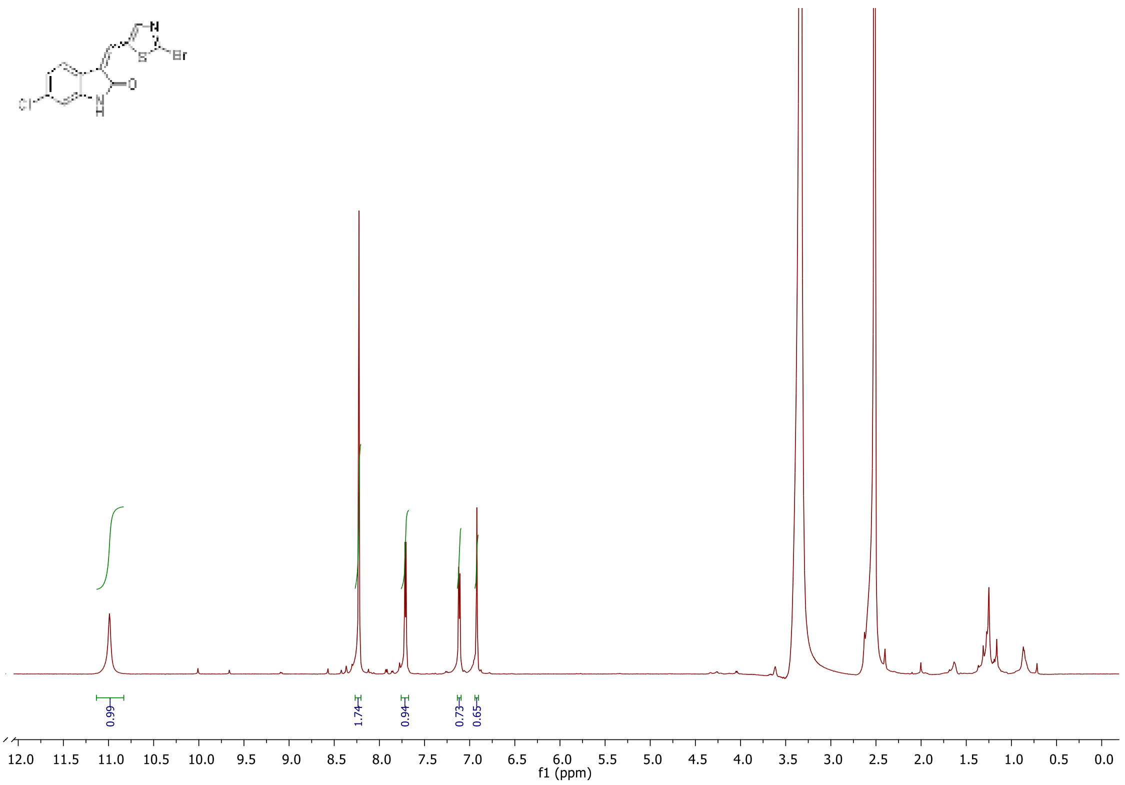

^{13}C NMR (150 MHz, DMSO-d_6) of **49**

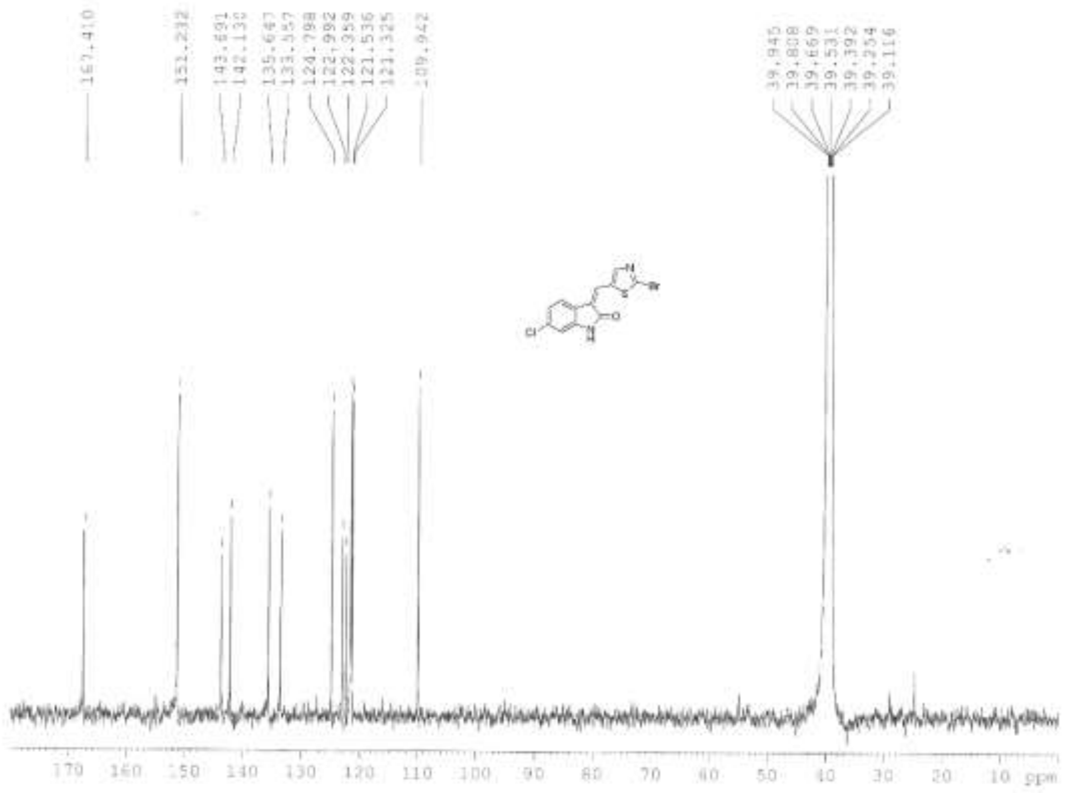

 Information Classification: General

^{1}H NMR (600 MHz, DMSO-d_6) of **50**

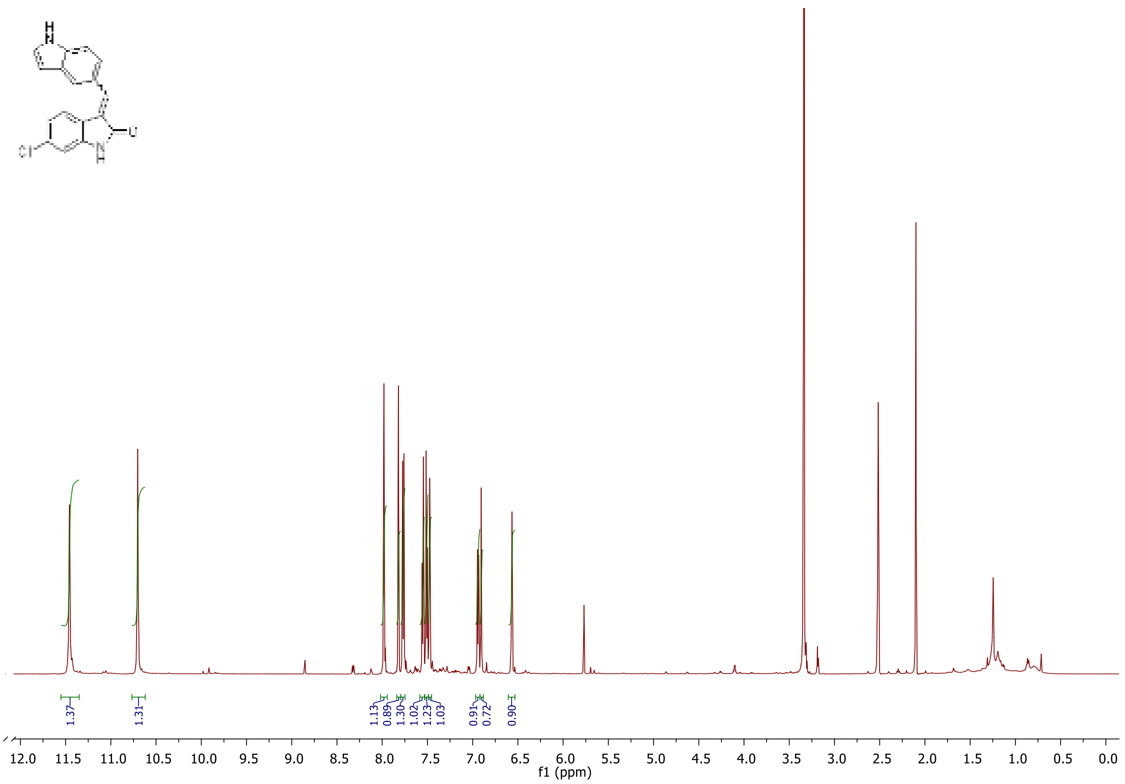

^{13}C NMR (150 MHz, DMSO-d_6) of **50**

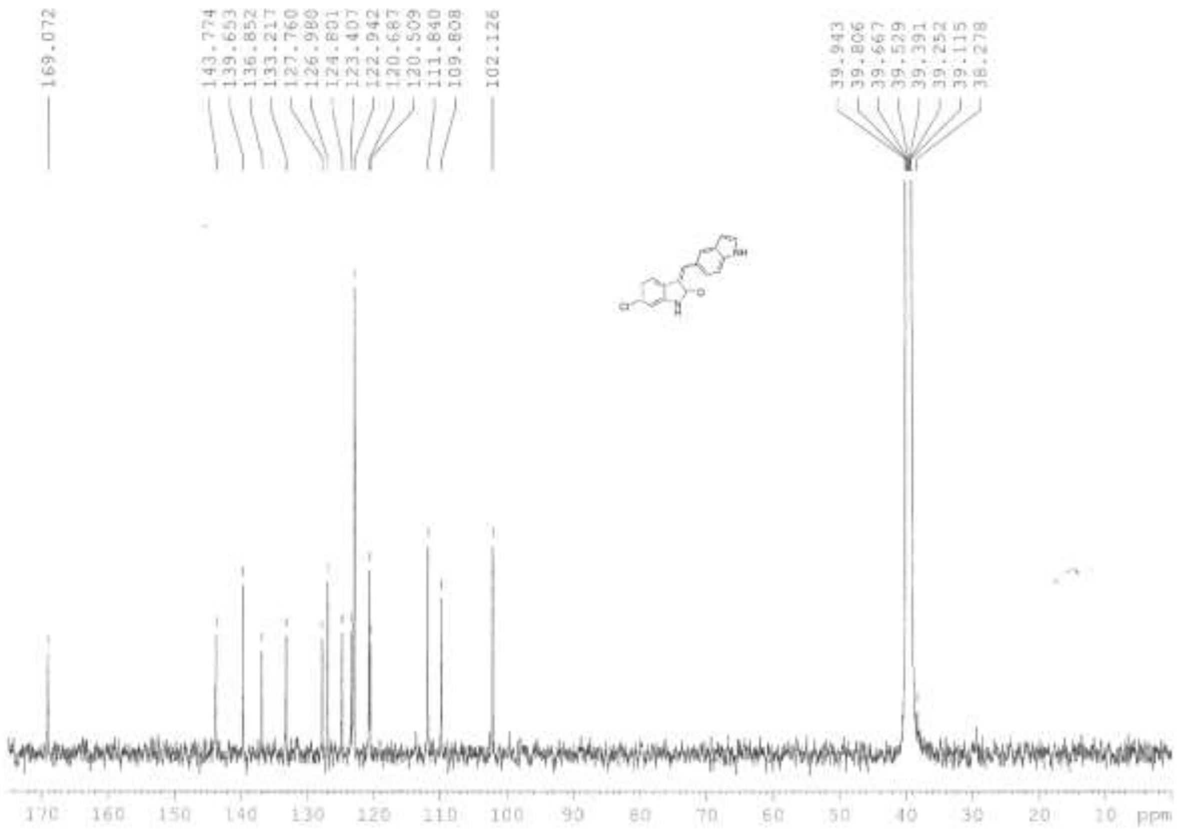

Information Classification: General
